# Insights into a novel class of azobenzenes incorporating 4,6-*O*-protected sugars as photo-responsive organogelators†

**DOI:** 10.1039/c9ra08033c

**Published:** 2019-12-19

**Authors:** P. V. Bhavya, V. Rabecca Jenifer, Panneerselvam Muthuvel, T. Mohan Das

**Affiliations:** Department of Chemistry, School of Basic and Applied Sciences, Central University of Tamil Nadu (CUTN) Thiruvarur 610005 India tmohandas@cutn.ac.in; Department of Organic Chemistry, University of Madras, Guindy Campus Chennai – 600025 India

## Abstract

A novel class of 4,6-*O*-butylidene/ethylidene/benzylidene β-d-glucopyranose gelator functionalized with photo-responsive azobenzene moieties were designed and synthesized and also characterized using different spectral techniques. These azobenzene-based organogelators can gel even at lower concentrations (critical gelation concentration – 0.5% and 1%). A morphological study of the gels shows one-dimensional aggregated bundles and helical fibres. The main driving force for the self-assembly is through cooperative interactions exhibited by the different groups *viz.*, sugar hydroxyl (hydrogen bonding interaction), azobenzene (aromatic π–π interaction) and alkyl chain of the protecting group (van der Waals interaction).

## Abstract 2

A novel class of 4,6-*O*-butylidene/ethylidene/benzylidene β-d-glucopyranose gelator functionalized with photo-responsive azobenzene moieties were designed and synthesized and also characterized using different spectral techniques.

## Graphical abstract

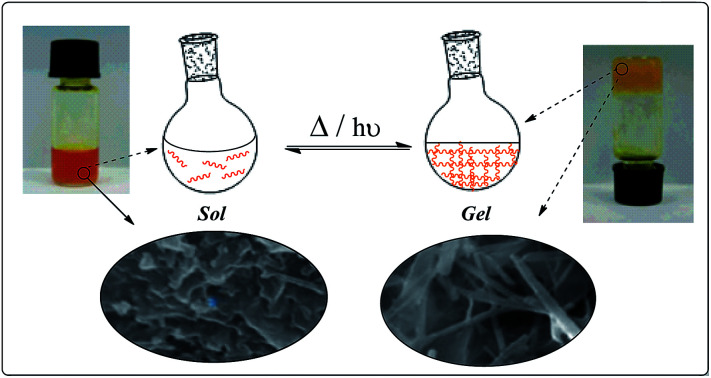

## 1. Introduction

**Fig. 1:**
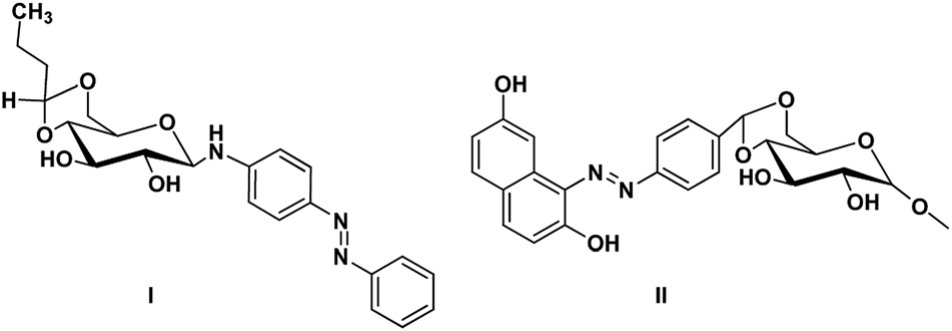
Representative examples of sugar-based low molecular weight organogelators reported in the literature.^1,13^

Gels are viscoelastic materials consisting of low molecular weight gelator molecules and solvents.^1^ Most of the gel formation takes place by multiple interactions, such as hydrogen bonding, π–π stacking, and hydrophobic interactions.^2^ Several class of organogelators have wide-range of applications as scaffolds for energy transfer,^3^ as drug delivery agents,^4^ self-healing materials,^5^ cosmetic additives,^6^ enzyme-immobilization matrices,^7^ oil recovery,^8^ tissue engineering^9^*etc.* Sugar-based gelators also have a significant role in many of these applications.^10^ One of the most interesting and also inspiring category of low molecular weight gelators (LMWGs), are multi-stimuli responsive supramolecular gelators (MRSGs). The self-assembly process of these smart materials are affected by some external stimuli such as heat, light, ultrasound, redox, and pH changes.^11^ LMWGs are an important class of soft matters and have received increasing attention in recent years due to their easy fabrication into soft materials,^12^ and also wide applications in the field of chemosensors^13^ drug delivery systems,^4^ optical devices,^14^ display devices, oil recovery, phase selective gelator^12^ removal of toxic dye^15^ and other applications.^16^ Carbohydrates and other polyhydroxy class of compounds are partly decorated with hydrophobic and hydrophilic moieties and were found to be one of the cheap and easy-to-synthesize LMWGs. The partial protection of carbohydrates as cyclic ketal derivatives are not only transports amphiphilicity but also supports in the preorganization by constraining the conformational freedom for self-assembly. Some of the ketal derivatives of carbohydrates have been reported as organogelators.^14^ Carbohydrates are hydrophilic building blocks that are able to form multiple H-bonds due to the presence of hydroxyl groups, which also favour solubilisation in water. Cyclic forms of carbohydrates provide more stable gels^17^ due to their directional hydroxyl groups which supports cooperative networks to have a self-assembled fibrous structure. Stability and gel properties are strongly dependent on the molecular structure of the gelator. The structural modification of carbohydrate molecules to obtain LMWGs has been an interesting field of research in recent years.^18^ Carbohydrate molecules are biocompatible; the gels derived from these molecules have wide application in biology and also as functional materials.^19^ Moreover, the abundant availability of saccharides enhances research into the design of novel sugar based gelator molecules I and II (Fig. 1). The incorporation of appropriate external photo responsive chromophores into gelator molecules leads to novel stimuli-responsive gels. For example, the introduction of azobenzene,^20^ stilbene,^21^ butadiene,^22^ dithienylethene,^23^ spiropyran,^24^ and anthracene groups^25^ are extensively incorporated to from photo-responsive materials. Similarly, the introduction of the *p*-methoxy group to the benzylidene acetal affects the gelation property. In the presence of acids, *p*-methoxybenzylidene acetal readily cleaves which is the resultant effect of pH and it triggered to release drug molecules.^26^ In general, the interactions of LMWGs are dynamic and reversible in nature. External stimuli on these gelators can affect the self-assembly of building blocks which is aggregated through multiple non-covalent interactions (such as hydrogen bonds, π–π stacking, electrostatic interactions, van der Waals forces). Depends on the functionalities of the gelator molecules, the gel–sol phase transitions have been triggered by pH value, temperature, mechanical stress, light, ionic species, host–guest interactions, redox processes, enzymes, *etc.*^27^

The soft materials fabricated from the azobenzene groups containing molecules can potentially be applied in message storage and increased charge transport process, *etc.*^28^ In several organogels, hydrophobic interactions such as aromatic π–π stacking and van-der Waals interactions are indispensable for the formation of oriented aggregates and are most probably the initial driving force for the self-assembly process. Furthermore, hydrogen bonding also seems to play a role in the orientation of molecules within the aggregates. Azobenzene-based cholesterol^29^ and aryl ethers^30^ were reported to possess gelating ability in organic solvents. In order to obtain a right balance between hydrophilic and hydrophobic nature of the gelator molecule, some of the hydrophilic groups like saccharide,^31^ peptide^32^ and PEG^33^ has been introduced to achieve the desire a hydrogelator property. In continuation to our on-going project in the area of the design and synthesis of sugar based gelators here we have reported the synthesis of sugar-azo derivatives and its organogelation properties. In order to obtain further insight into the structure–function relationships of sugar-based organogelators, we have constructed a small library of sugar derivatives that contain aromatic moieties (*i.e.*, azobenzene) linked to the saccharide moiety through *N*-glycosyl bridges.

## 2. Result and discussion

### 2.1. Synthesis and characterization of sugar-azo derivatives

**Scheme 1:**
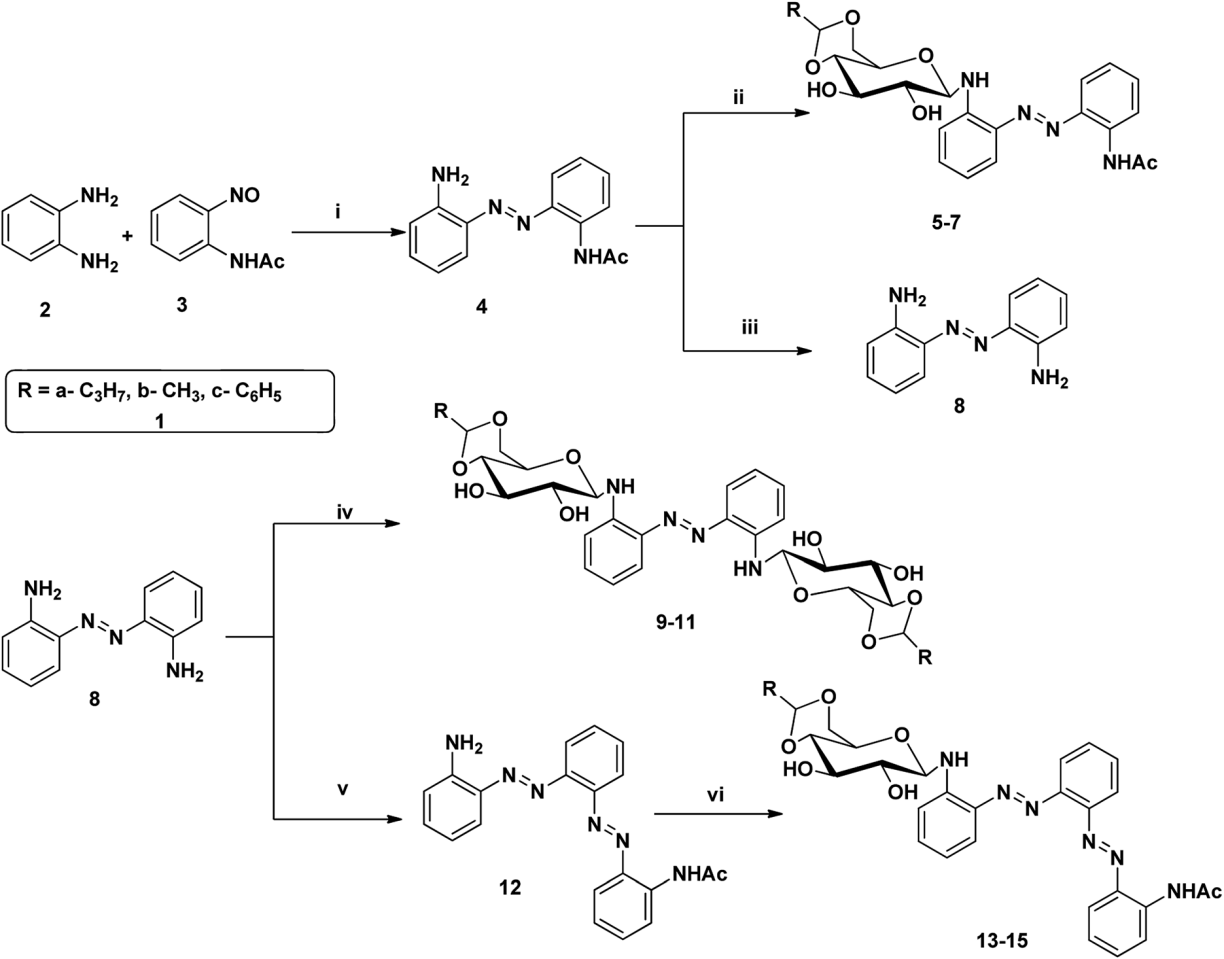
Synthesis of sugar-azo derivatives 5–7, 9–11 & 13–15. Reagents and reaction conditions: (i) toluene, AcOH, 60 °C, 21 h, 70% (ii) 4,6-*O*-protected d-glucose, 81–88% 1(a–c), ethanol, rt, (iii) KOH, 90 °C, 1 h, 94% (iv) 4,6-*O*-protected d-glucose 1(a–c), ethanol, rt, 71–83% (v) compound 3, toluene, AcOH, 60 °C, 16% (vi) 4,6-*O*-protected d-glucose 1(a–c), ethanol, rt, 66–85%.

Azobenzene based *N*-glycosylamine derivatives (5–7, 9–11 & 13–15) were synthesized from amino azobenzene derivatives (4, 8, 12)^34^ and 4,6-*O*-protected-d-glucose (1a–c) (Scheme 1).^35,36^ The reactants were chosen preferentially to have a desired functional groups, such as the primary amine in the aromatic moiety and active hydroxyl group in the 4,6-*O*-protected-d-glucose. All the new resulting azobenzene *N*-glycosylamine compounds (5–7, 9–11 & 13–15) were identified through spectral techniques. During the synthesis of different *N*-glycosylamines using partially protected saccharide,^37^ gel formation were observed and this observation prompted us to go for the study of the gelation property of azobenzene containing saccharide compounds (5–7, 9–11 & 13–15). The identities of all the synthesized *N*-glycosylamines were confirmed using NMR (^1^H and ^13^C) and elemental analysis. All the synthesized compounds were subjected to gelation studies with a wide range of solvents and the gels thus obtained were characterized using microscopic techniques, *viz.*, SEM and HR-TEM analysis.

Using ^1^H NMR the presence of acetyl methyl proton was confirmed as sharp singlet in the region of 1.88–2.90 ppm. The protons of the glucose moiety resonate at around 3.00–5.60 ppm, the β-anomeric form of the glycosidic unit was confirmed by the triplet in the range of 4.41–4.90 ppm with a coupling constant between 4.8–6.6 Hz (ref. 38) (Table 1). The NH proton appears as a doublet in the region of 4.03–4.54 ppm with a coupling constant between 4.8–6.9 Hz (see ESI for further details†).

**Table 1:** Synthesis of azobenzene based *N*-glycosylamines, (5–7, 9–11 & 13–15)

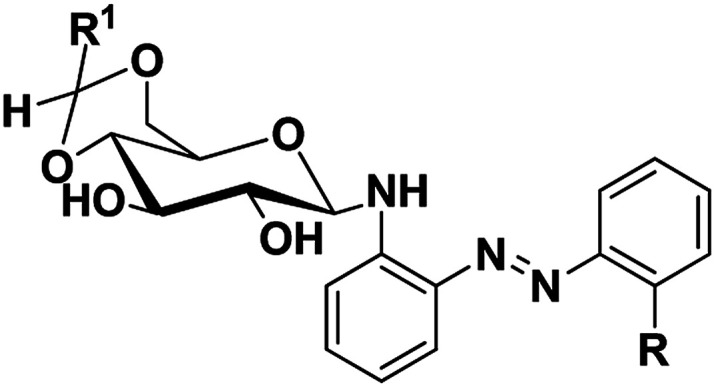				
Compd no.	Structure of the compound	*δ* (ano-H), ^3^*J*_H1_,_H2_/Hz	*δ* (gly-NH), ^3^*J*_H1_,_H2_/Hz	Yield (%)
5	R^1^=C_3_H_7_	4.90, 5.1	4.51, 5.4	82
	R=NHAc			
6	R^1^=CH_3_	4.43, 5.1	4.24, 7.8	81
	R=NHAc			
7	R^1^=C_6_H_5_	4.64, 6.6	4.03, 4.8	88
	R=NHAc			
9	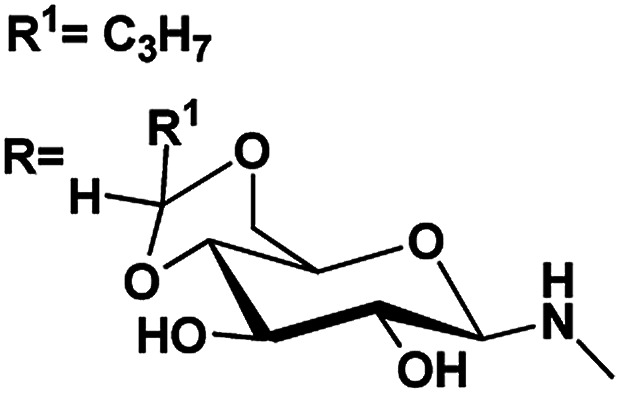	4.82, 4.8	4.18, 6.9	71
10	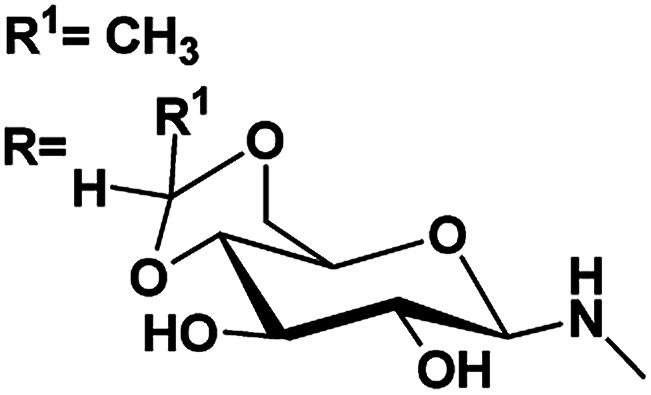	4.72, 4.8	4.54, 5.7	78
11	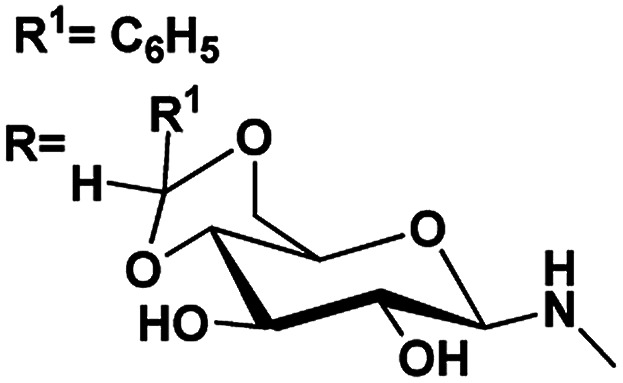	4.97, (—)^a^	4.38, (—)^a^	83
13	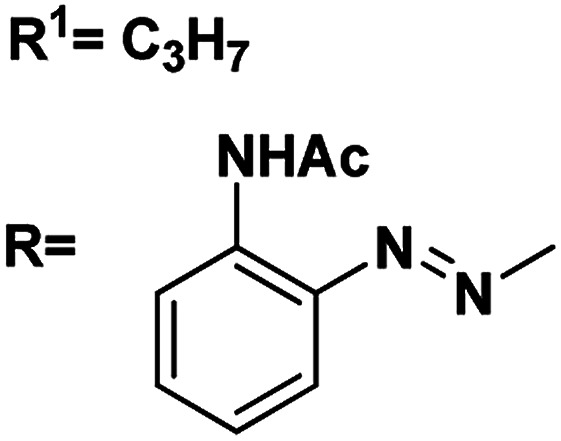	4.54, 5.1	4.10, 4.8	66
14	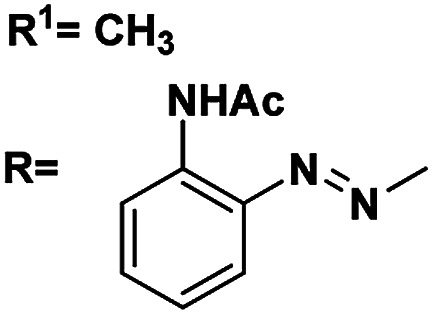	4.73, 4.8	4.41, (—)^a^	76
15	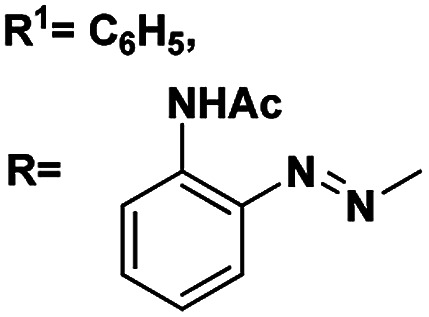	4.59, (—)^a^	4.15, (—)^a^	85

a Peaks overlapped with saccharide protons.

From ^13^C NMR analysis of compounds (5–7, 9–11 & 13–15) the methyl carbon atoms of the acetyl group appear in the range of 22.1–29.6 ppm. 107.0 ppm corresponds to acetal carbon of ethylidene, benzylidene and butylidene derivatives, respectively. The presence of azobenzene moiety can be confirmed from the appearance of the peaks in the range of 107.0–150.0 ppm. In addition, the presence of the carbonyl carbons was identified from the appearance of the peak at 168.0–174.0 ppm.

### 2.2. Organogel formation

**Fig. 2:**
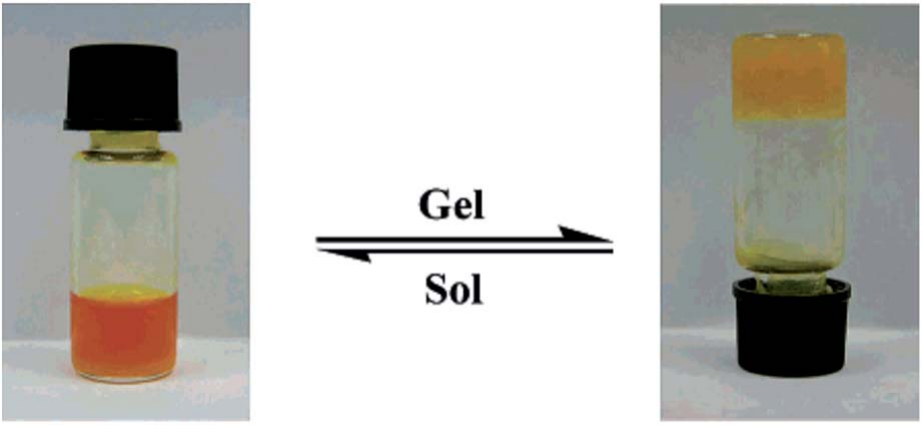
Picture of sol–gel transition of compound 13 in *p*-xylene (0.5 mg mL^−1^).

The gelation ability of these compounds in different solvents was determined by “stable to inversion of the container” method.^39^ A weighed amount of the corresponding azo-benzene based *N*-glycosylamines (5 mg) in 1 mL of an organic solvent was heated in a septum-capped test tube until the solid dissolved; the resultant mixture was then cooled to room temperature, and when the tube could be inverted without any flow, it was determined to be a “gel” (Fig. 2). Furthermore, critical gelation concentration (CGC) refers to the concentration at which a minimum amount of compound forms a gel (Table 2). The more gelating ability of butylidene protected *N*-glycosylamines (5, 9 & 13), seems to be due to the presence of the long alkyl chain in these compounds (in ethylidene only one –CH_3_ group is present), as a result of higher van der Waals force^40^ and such interactions are expected to be less in ethylidene protected *N*-glycosylamines (6, 10 & 14) and the corresponding gelation would be low.

**Table 2:** Gelation studies of azo-benzene based *N*-glycosylamines (5–7, 9–11 & 13–15)

Status of compound^a^ (CGC%)									
Solvent	5	6	7	9	10	11	13	14	15
CHCl_3_	S	S	S	S	S	S	S	S	S
DCM	S	S	S	S	S	S	S	S	S
Hexane	I	I	I	I	I	I	I	I	I
EtOH	P	P	P	P	P	P	P	P	P
MeOH	P	P	P	P	P	P	P	P	P
DMF	S	S	S	S	S	S	S	S	S
THF	P	P	P	P	P	P	P	P	P
EtOAc	S	S	S	S	S	S	S	S	S
Toluene	PG	PG	PG	PG	PG	PG	PG	PG	PG
*p*-Xylene	G (0.5)	G (1.5)	G (1)	G (0.5)	G (1.5)	G (1)	G (0.5)	G (1.5)	G (1)
*m*-Xylene	G (1)	G (1.5)	G (1)	G (0.5)	G (2)	PG	G (0.5)	G (1.5)	G (1)
*o*-Xylene	PG	PG	G (0.5)	G (1)	G (1)	G (1)	G (1)	G (2)	PG
Benzene	G (0.5)	G (2)	G (1)	G (0.5)	G (2)	PG	PG	G (2)	G (1)
NO_2_-benzene	G (0.5)	PG	PG	G (0.5)	G (1)	G (0.5)	G (0.5)	G (2)	PG

a G – gelation, PG – partial gelation, S – soluble, I – insoluble, P – precipitation, CGC – Critical Gelation Concentration.

### 2.3. Sol–gel transition

The benzylidene protected *N*-glycosylamines (7, 11 & 15) and the corresponding gelation would be moderate. However, in methyl substituted *N*-glycosylamines (7, 11 & 15) the steric interactions seem to be largely responsible for hampering the gelation properties. These results support that alkyl group present at *ortho* position influences the formation of intermolecular H-bonding which is responsible for gelation process in partially protected *N*-glycosylamines. The presence of significant involvement of van der Waals forces and the structural arrangement that enhances the π–π interactions are some of the factors responsible for good gelation abilities. Among the various polar and nonpolar solvents used for gelation of *N*-glycosylamines (5–7, 9–11 & 13–15) benzene, *p*-xylene, *m*-xylene, *o*-xylene and nitrobenzene were found to be best solvents for the gelation process which may be attributed to a strong solute–solvent interaction.

### 2.4. Morphological studies

**Fig. 3:**
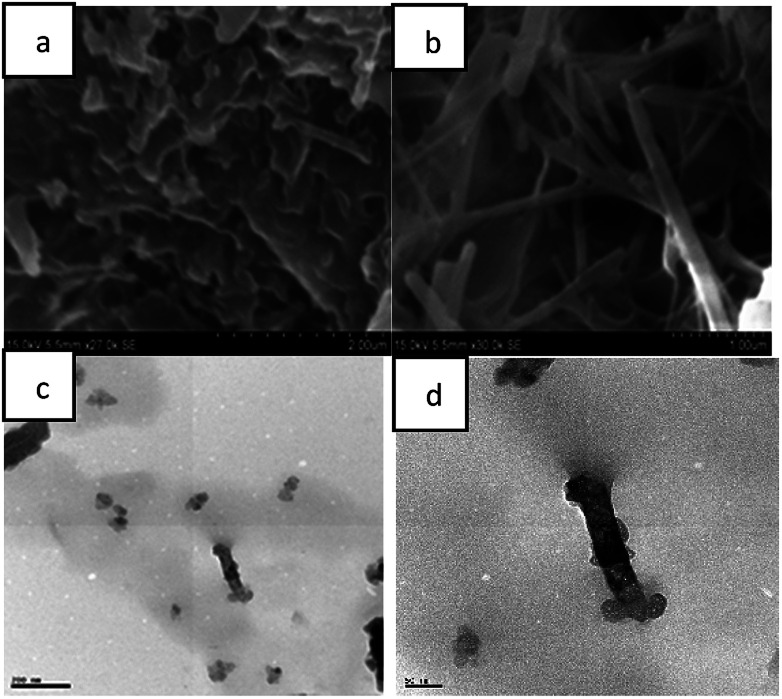
SEM and HR-TEM images of compound, 13 from *p*-xylene: (a) (0.5% w/v *p*-xylene) solution; (b–d) gel.

The morphology of azo-benzene based *N*-glycosylamines in the solution and gel states were studied with SEM and HR-TEM analysis (Fig. 3).

SEM image (Fig. 3a) of *N*-glycosylamine 13, recorded immediately after heating the compound 13 (0.5% w/v) in *p*-xylene, shows a bundles-like structure which is due to the identical state of the *N*-glycosylamine molecules in solution state. However, cooling the solution to room temperature and led to the formation of a gel, which shows a nano-fibrous network (Fig. 3b). Therefore the SEM analysis revealed the conversion of bundle-like structure into nano-fibrous upon self-assembly in the gel state^41^ and HR-TEM image of compound 13 also showed fibers network (Fig. 3c and d). The SEM and HR-TEM analysis clearly indicates the formation of gels due to self-assembly of the molecules.

### 2.5. Thermal analysis

**Fig. 4:**
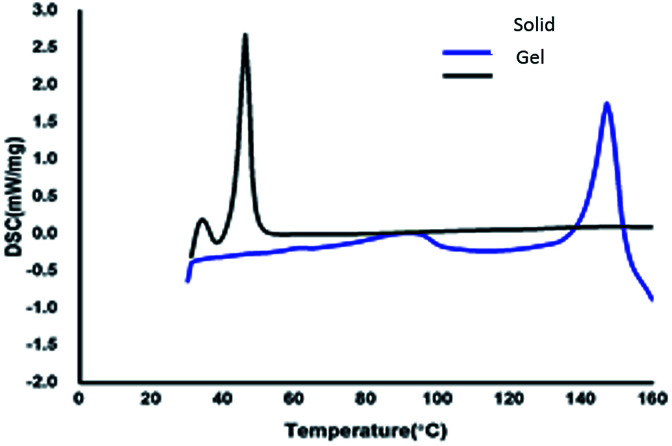
DSC spectra of compound 13 and its gel in *p*-xylene.

To understand the thermal properties of compound 13, the comparative study of the gelator in the solid and xerogel states were studied using differential scanning calorimetric (DSC) technique. DSC graphs of azobenzene based *N*-glycosylamine 13 and its xerogel are shown in Fig. 4*p*-xylene gel (0.5%) was utilized for DSC studies, upon heating the gel at the rate of 10 min^−1^ the evaporation of solvent will take place and result in formation of the xerogel. The solid and the xerogel of *N*-glycosylamine 13 show the phase transition at 152.10 °C (74.2 J g^−1^) and 46.10 °C (116.9 J g^−1^) respectively. From the DSC graph it was evidenced that *N*-glycosylamine 13 is thermally more stable in the xerogel state than in the solid state. This is due to the ordered arrangement of gelator in the self-assembled state and which is not available in the solid state.^42^ The phase transition temperature of compound 13 and its corresponding gel are *T*_g_s (°C) 147 and Δ*H* (J g^−1^) 116. The melting temperature of the gelator and gel are obtained from DSC experiments.

### 2.6. Powder XRD analysis

**Fig. 5:**
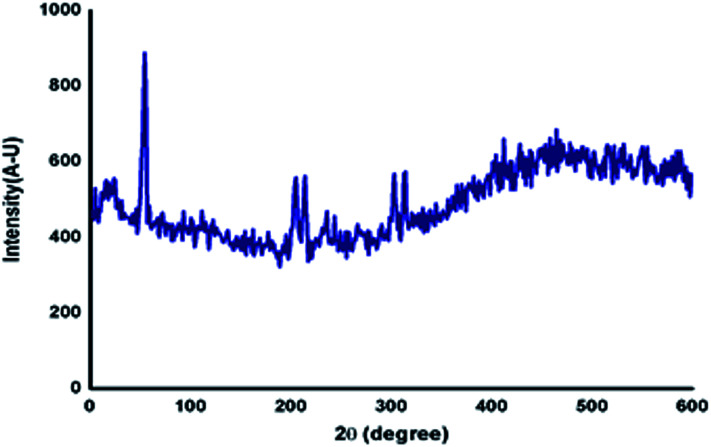
Powder XRD diffraction of xerogel, 13 (*p*-xylene 0.5%).

Generally, the self-assembly of the molecules in the gel state was studied using powder X-ray diffraction studies of the xerogel.^43^ The gel of azobenzene *N*-glycosylamine, 13 was prepared at the concentration of 0.5% in *p*-xylene and allowed to stand at ambient temperature to obtain the xerogel. From the XRD (Fig. 5) pattern, it was confirmed that the self-assembly of gelator 13 was due to van der Waals force between the alkyl chain and π–π interaction between the azobenzene core. The strong diffraction at a low *θ* angle of 6.969 corresponds to van der Waals force and a broad peak at 25.761 arises from π–π interaction of the azobenzene unit. Peaks at *θ* = 6.002, 9.351, 12.811, 13.551, 14.384, 16.901, 17.633 and 23.398 show the crystalline nature of the gel fibers.

### 2.7. Absorption studies

**Fig. 6:**
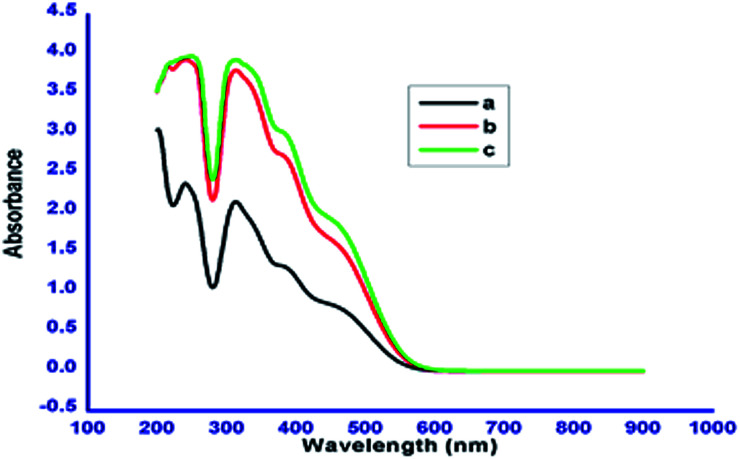
Absorption spectra of compounds 12 (a), 13 (b) and 15 (c) in acetonitrile (1 × 10^−5^ M).

The chromophoric nature of the azobenzene based *N*-glycosylamines 12, 13 & 15 was investigated using absorption spectroscopy. The absorption spectra of *N*-glycosylamines have been recorded at the concentration of 1 × 10^−5^ M in acetonitrile. The compounds 12, 13 & 15 show characteristic absorption bands at around 279 nm and 440 nm (Fig. 6). The change in the number of methylene units in the alkyl chain and the 4, 6-*O*-protecting group in the d-glucose unit does not influence the absorption maxima.

## 3. Experimental section

### 3.1. Materials and methods

d-Glucose and *O*-phenylene diamine were purchased from Sd-fine, India. Benzaldehyde, dimethyl acetal was purchased from Sigma Aldrich chemicals Pvt. Ltd, USA. Paraldehyde, potassium hydroxide, acetic anhydride, acetic acid and butyraldehyde were purchased from SRL, India. Toluene and ethanol were used after distillation. Column chromatography was performed on silica gel (100–200 mesh). NMR spectra were recorded on a Bruker DRX 300 MHz, spectrometer. Elemental analysis was performed by using PerkinElmer 2400 series CHNS/O analyser. The gels were imaged with a HITACHI-S-3400W Scanning Electron. Thermal transitions for gelators and gels were determined on a NETZSCH DSC 204 instrument. Diffractograms of the dried films were recorded on XRD RINT 2500 diffractometer using Ni filtered Cu Kα radiation X-ray diffractograms of the dried films were recorded on XRD RINT 2500 diffractometer using Ni filtered Cu Kα radiation.

### 3.2. Synthesis of 2-acetamido-2′-amino-*ortho*-azobenzene (4)

Compound, 4 was prepared according to literature procedures^33^ from *o*-phenylenediamine 2 (0.821 g, 7.58 mmol) and 2-nitrosoacetanilide 3 (1.25 g, 7.71 mmol).^43^ The residue was purified by flash column chromatography (SiO_2_, hexane/EtOAc, 1 : 1), and it was obtained as red solid in 70% yield (1.35 g): mp 148–150 °C (lit. 143 °C); ^1^H NMR (300 MHz, CDCl_3_) *δ* 9.95 (s, 1H), 8.63 (d, *J* = 8.3 Hz, 1H), 7.70 (dd, *J* = 8.1, 1.6 Hz, 1H), 7.62 (dd, *J* = 8.1, 1.3 Hz, 1H), 7.45–7.37 (m, 1H), 7.29–7.22 (m, 1H), 7.18–7.10 (m, 1H), 6.87–6.71 (m, 2H), 5.40 (s, 2H), 2.27 (s, 3H); ^13^C NMR (75 MHz, CDCl_3_) *δ* 168.6, 145.0, 137.5, 135.5, 133.2, 131.8, 124.4, 123.5, 122.2, 120.5, 120.2, 118.0, 117.4, 25.5. HRMS (ESI) *m*/*z* calcd [M + Na]^+^: 277.10517 found 277.10598.

### 3.3. Synthesis of 2,2′-diamino-*ortho*-azobenzene (8)

Compound, 8 was prepared according to the literature procedures.^33^ A solution of 2-acetamido-2′-amino-*ortho*-azobenzene 4 (740 mg, 2.91 mmol) in 138 mL of ethanol was treated with a solution of KOH (12.1 g, 215 mmol) in 80 mL of ethanol and 32 mL of water. The mixture was heated to 90 °C. After 1 h, the mixture was poured into 500 g of ice and extracted with CH_2_Cl_2_. It was dried over Na_2_SO_4_ and concentrated to yield 0.583 g (94%): mp 136–138 °C; ^1^H NMR (300 MHz, CDCl_3_) *δ* 7.68 (dd, *J* = 8.0, 1.4 Hz, 2H), 7.21–7.14 (m, 2H), 6.83–6.74 (m, 4H), 5.49 (s, 4H). The analytical data corresponds to the literature.^37^ HRMS (ESI) *m*/*z* calcd [M + H]^+^: 213.11275 found 213.11347.

### 3.4. Synthesis of 2-acetamido-2′′-amino-*ortho*-bisazobenzene (12)

Compound, 12 was prepared according to the literature procedures^33^ from a solution of 2,2′-diaminodiazobenzene 8 (300 mg, 1.41 mmol) and 2-nitrosoacetanilide 3 (230 mg, 1.41 mmol). After 2.5 days, the solvent was removed under reduced pressure, and the residue was purified by flash column chromatography (SiO_2_, hexane/EtOAc, 2 : 1). The product was obtained as a red solid (80.3 mg, 16%): mp 158–162 °C; ^1^H NMR (300 MHz, CDCl_3_) *δ* 10.12 (s, 1H), 8.69 (d, *J* = 8.3 Hz, 1H), 7.88 (dd, *J* = 8.1, 1.5 Hz, 1H), 7.85 (dd, *J* = 8.1, 1.5 Hz, 1H), 7.81 (dd, *J* = 7.9, 1.5 Hz, 1H), 7.72 (dd, *J* = 7.7, 1.6 Hz, 1H), 7.62–7.44 (m, 3H), 7.20 (dd, *J* = 15.3, 8.4, 1.4 Hz, 2H), 6.87–6.81 (m, 1H), 6.74 (dd, *J* = 8.2, 1.0 Hz, 1H), 6.31 (s, 2H), 2.00 (s, 3H). The analytical data correspond to the literature.^37^ HRMS (ESI) *m*/*z* calcd [M + Na]^+^: 381.14265 found 381.14343.

### 3.5. General procedure for the synthesis of *N*-glycosylamines (5–7, 9–11 & 13–15)

To a solution of 1.0 mmol of the 4,6-*O*-protected-β-d-glucopyranose 1a–c in 10 mL of ethanol, was added 1.2 mmol of the azo-benzene based amine 4, 8, 12. The reaction was then stirred at room temperature, the reactants dissolved within 5–10 minutes. Progress of the reaction was monitored by TLC, which was then filtered and dried.

#### 3.5.1. Physicochemical and spectral data for 4,6-*O*-butylidene-*N*-(2-acetamido-2′-amino-*ortho*-azobenzyl)-β-d-glucopyranosyl amine, 5

Compound, 5 was obtained by the reaction of 2-acetamido-2′-amino-*ortho*-azobenzene 4 (1.2 mmol, 0.65 g) and 4,6-*O*-butylidene-β-d-glucopyranose 1a (1.0 mmol, 0.5 g) as a red solid. Yield: 0.82 g (82%); mp 140–143 °C; ^1^H NMR (CDCl_3_ + DMSO-d_6_, 300 MHz), *δ* 8.61 (d, *J* = 6 Hz, 1H, Ar-H), 7.96–8.03 (m, 2H, Ar-H), 7.58 (t, 1H, Ar-H), 7.47 (m, 1H, Ar-H), 7.27–7.30 (d, *J* = 9 Hz, 1H, Ar-H), 7.12–7.18 (m, 1H, Ar-H), 6.09 (s, 2H, Ar-H), 5.67 (s, 1H, Ace-H), 5.19 (d, *J* = 3 Hz, 1H, Sac-H), 5.09 (t, *J* = 3 Hz, 1H, Sac-H), 4.90 (t, *J* = 6 Hz, 1H, Ano-H), 4.51 (d, *J* = 6 Hz, 1H, –NH), 3.81–3.84 (m, 2H, Sac-H), 3.66 (s, 2H, Sac-OH), 2.90 (s, 2H, Sac-H), 2.63 (s, 3H, –NHCOCH_3_), 1.97 (t, *J* = 4.5 Hz, 2H, Sac-CH_2_), 1.77 (m, 2H, Sac-CH_2_), 0.45 (s, 3H, Sac-CH_3_). ^13^C NMR (CDCl_3_ + DMSO-d_6_, 75 MHz) *δ* 173.6, 149.8, 141.8, 139.9, 138.0, 137.7, 135.8, 128.3, 127.7, 125.6, 124.3, 122.1, 121.9, 107.1, 85.0, 79.2, 78.1, 73.4, 73.1, 67.0, 35.7, 29.8, 22.1, 18.7. Anal. calcd for C_24_H_30_N_4_O_6_: C, 61.26; H, 6.43; N, 11.91. Found: C, 60.54; H, 6.33; N, 11.85. HRMS (ESI) *m*/*z* calcd [M + Na]^+^: 493.20544 found 493.20516.

#### 3.5.2. Physicochemical and spectral data for 4,6-*O*-ethylidene-*N*-(2-acetamido-2′-amino-*ortho*-azobenzyl)-β-d-glucopyranosyl amine, 6

Compound, 6 was obtained by the reaction of 2-acetamido-2′-amino-*ortho*-azobenzene 4 (1.2 mmol, 0.73 g) and 4,6-*O*-ethylidene-β-d-glucopyranose 1b (1.0 mmol, 0.5 g) as a red solid. Yield: 0.50 g (81%); mp 142–144 °C; ^1^H NMR (CDCl_3_ + DMSO-d_6_, 300 MHz), *δ* 8.89 (d, *J* = 8.1 Hz, 1H, Ar-H), 8.09 (d, *J* = 7.8 Hz, 1H, Ar-H), 8.03 (d, *J* = 8.4 Hz, 1H, Ar-H), 7.84 (s, 1H, Ar-H), 7.76 (t, *J* = 7.8 Hz, 1H, Ar-H), 7.50–7.63 (m, 2H, Ar-H), 7.23 (d, *J* = 8.1 Hz, 1H, –NH), 7.13 (t, *J* = 7.5 Hz, 1H, Ace-H), 5.54 (s, 1H, Sac-H), 4.40–4.51 (m, 2H, Sac-H), 4.43 (t, *J* = 5.1 Hz, 1H, Ano-H), 4.24 (t, *J* = 7.8 Hz, 1H, –NH), 3.87 (d, *J* = 12 Hz, 2H, Sac-H), 3.85–3.94 (m, 2H, Sac-H), 3.62–3.70 (m, 2H, Sac-OH), 2.63 (s, 3H, –NHCOCH_3_), 1.72 (d, *J* = 6 Hz, 3H, Sac-CH_3_). ^13^C NMR (CDCl_3_ + DMSO-d_6_, 75 MHz) *δ* 177.07, 148.98, 146.66, 142.52, 142.18, 136.44, 136.32, 129.86, 127.48, 123.06 (2C), 121.86, 118.30, 107.07, 90.36, 85.19, 79.26, 79.01, 73.14, 72.26, 41.01, 22.19. Anal. calcd for C_22_H_26_N_4_O_6_: C, 59.72; H, 5.92; N, 12.66. Found: C, 59.54; H, 5.90; N, 12.55. HRMS (ESI) *m*/*z* calcd [M + Na]^+^: 465.17401 found 465.17446.

#### 3.5.3. Physicochemical and spectral data for 4,6-*O*-benzylidene-*N*-(2-acetamido-2′-amino-*ortho*-azobenzyl)-β-d-glucopyranosyl amine, 7

Compound, 7 was obtained by the reaction of 2-acetamido-2′-amino-*ortho*-azobenzene 4 (1.2 mmol, 0.56 g) and 4,6-*O*-benzylidene-β-d-glucopyranose 1c (1.0 mmol, 0.5 g) as a red solid. Yield: 0.40 g (88%); mp 140–143 °C; ^1^H NMR (CDCl_3_ + DMSO-d_6_, 300 MHz), *δ* 8.69 (d, *J* = 8.4 Hz, 1H Ar-H), 7.91 (d, *J* = 8.1 Hz, 1H, Ar-H), 7.64–7.73 (m, 2H, Ar-H), 7.48 (d, *J* = 5.4 Hz, 2H, Ar-H), 7.15–7.21 (m, 2H, Ar-H), 6.85 (d, *J* = 8.4 Hz, 3H, Ar-H), 6.74 (t, *J* = 8.1 Hz, 2H, Ar-H), 5.52 (s, 2H, Ace-H), 5.1 (s, 1H, Sac-H), 4.64 (t, *J* = 6.6 Hz, 1H, Ano-H), 4.20–4.30 (m, 2H, Sac-H), 3.91–4.02 (d, *J* = 4.8 Hz, 1H, –NH), 3.70–3.75 (m, 2H, Sac-OH), 3.44–3.51 (m, 3H, Sac-H), 2.50 (s, 3H, –NHCOCH_3_). ^13^C NMR (CDCl_3_ + DMSO-d_6_, 75 MHz) *δ* 173.6, 150.0, 141.6, 140.0 (2C), 137.6, 135.5, 133.7, 132.8 (2C), 131.3 (2C), 127.8, 126.4, 123.5, 122.1, 121.5, 120.6 (2C), 106.4, 98.6, 86.6, 80.8, 73.9, 71.1, 66.9, 29.6. Anal. calcd for C_27_H_28_N_4_O_6_: C, 64.27; H, 5.59; N, 11.10. Found: C, 64.10; H, 5.20; N, 11.05.

#### 3.5.4. Physicochemical and spectral data for 4,6-*O*-butylidene-*N*-(2,2′-diamino-*ortho*-azobenzyl)-β-d-glucopyranosyl amine, 9

Compound, 9 was obtained by the reaction of 2,2′-diamino-*ortho*-azobenzene 8 (0.50 mmol, 0.3 g) and 4,6-*O*-butylidene-β-d-glucopyranose 1a (1.0 mmol, 0.54 g) as a red solid. Yield: 0.50 g (71%); mp 140–142 °C; ^1^H NMR (CDCl_3_ + DMSO-d_6_, 300 MHz), *δ* 7.62 (d, *J* = 9.0 Hz, 2H Ar-*H*), 7.12 (t, *J* = 7.5 Hz, 2H, Ar-*H*), 6.80 (d, *J* = 7.8 Hz, 3H, Ar-*H*), 6.70 (t, *J* = 7.5 Hz, 1H, –N*H*), 6.29 (s, 1H, Ar-*H*), 5.99 (s, 2H, Ace-*H*), 4.82 (d, *J* = 4.8, 2H, Ano-*H*), 4.74–5.12 (m, 3H, Sac-*H*), 4.55 (d, *J* = 11.4 Hz, 4H, Sac-*H*), 4.00–4.19 (m, 3H, Sac-H), 4.18 (d, *J* = 6.9 Hz, 1H, –N*H*), 3.59 (d, *J* = 9.3 Hz, 2H, Sac-*H*), 3.41–3.56 (m, 4H, Sac-OH), 1.58 (d, *J* = 4.8 Hz, 4H, Sac-C*H*_2_), 1.37–1.44 (m, 4H, Sac-CH_2_), 0.92 (t, *J* = 7.5 Hz, 6H, Sac-CH_3_). ^13^C NMR (CDCl_3_ + DMSO-d_6_, 75 MHz) *δ* 142.7, 130.2, 123.1, 116.1, 115.85 101.2, 96.6, 92.1, 80.1, 77.1, 75.0, 72.4, 69.6, 35.3, 16.4, 13.0. Anal. calcd for C_32_H_44_N_4_O_10_: C, 59.61; H, 6.88; N, 8.69. Found: C, 58.60; H, 6.60; N, 8.50. HRMS (ESI) *m*/*z* calcd [M + Na]^+^: 667.29244 found 667.29496.

#### 3.5.5. Physicochemical and spectral data for 4,6-*O*-ethylidene-*N*-(2,2′-diamino-*ortho*-azobenzyl)-β-d-glucopyranosyl amine, 10

Compound, 10 was obtained by the reaction of 2,2′-diamino-*ortho*-azobenzene 8 (0.50 mmol, 0.3 g) and 4,6-*O*-ethylidene-β-d-glucopyranose 1b (1.0 mmol, 0.48 g) as a red solid. Yield: 0.50 g (78%); mp 141–143 °C; ^1^H NMR (CDCl_3_ + DMSO-d_6_, 300 MHz), *δ* 8.23 (d, *J* = 6.0 Hz, 1H Ar-H), 7.61–7.83 (m, 3H, Ar-H), 7.10–7.20 (m, 2H, Ar-H), 6.98 (d, *J* = 8.4 Hz, 2H, Ar-H), 6.69 (t, *J* = 7.8 Hz, 1H, Aro-H), 6.42 (s, 2H, Ace-H), 5.03 (d, *J* = 6.8 Hz, 3H, Sac-H), 4.90 (s, 2H, Sac-H), 4.72 (t, *J* = 4.8 Hz, 1H, Ano-H), 4.54 (t, *J* = 5.7 Hz, 1H, –NH), 3.98–4.09 (m, 2H, Sac-H), 3.77–3.82 (m, 4H, Sac-OH), 3.19–3.3.26 (m, 4H, Sac-H), 1.33 (t, *J* = 4.8 Hz, 6H, Sac-CH_3_). ^13^C NMR (CDCl_3_ + DMSO-d_6_, 75 MHz) *δ* 139.9, 137.7, 135.8, 128.3, 124.2 (2C), 104.1, 97.8, 85.6, 80.7, 78.1, 75.4, 66.8, 29.8. Anal. calcd for C_28_H_36_N_4_O_10_: C, 57.13; H, 6.16; N, 9.52. Found: C, 56.80; H, 5.90; N, 8.90. HRMS (ESI) *m*/*z* calcd [M − H]^+^: 588.24314 found 587.23621.

#### 3.5.6. Physicochemical and spectral data for 4,6-*O*-benzylidene-*N*-(2,2′-diamino-*ortho*-azobenzyl)-β-d-glucopyranosyl amine, 11

Compound, 11 was obtained by the reaction of 2,2′-diamino-*ortho*-azobenzene 8 (0.50 mmol, 0.3 g) and 4,6-*O*-benzylidene-β-d-glucopyranose 1c (1.0 mmol, 0.63 g) as a red solid. Yield: 0.65 g (83%); mp 142–144 °C; ^1^H NMR (CDCl_3_ + DMSO-d_6_, 300 MHz), *δ* 7.60 (d, *J* = 8.1 Hz, 2H, Ar-H), 7.50 (d, 2H, Ar-H), 7.47 (d, *J* = 3.9 Hz, 4H, Ar-H), 7.32 (t, *J* = 6 Hz, 4H, Ar-H), 7.11 (t, *J* = 6.9 Hz, 1H, Ar-H), 6.93 (s, 2H, Ar-H), 6.80 (d, *J* = 8.1 Hz, 1H, Ar-H), 6.68 (t, *J* = 7.2 Hz, 1H, Ar-H), 6.45 (s, 1H, Ar-H), 6.05 (s, 1H, Ace-H), 5.10 (d, *J* = 7.8 Hz, 2H, –NH), 4.97 (s, 2H, Ano-H), 4.59 (d, *J* = 3.3 Hz, 1H, Sac-H), 4.38 (s, 1H, –NH), 4.15–4.27 (m, 5H, Sac-H), 3.86 (t, *J* = 8.4 Hz, 2H, Sac-H), 3.63–3.94 (m, 6H, Sac-H, Sac-OH), 3.48 (s, 2H, Sac-H). ^13^C NMR (CDCl_3_ + DMSO-d_6_, 75 MHz) *δ* 136.8, 136.7, 128.0 (2C), 127.1 (2C), 125.6 (2C), 125.6, 123.0, 100.7, 100.6, 92.3, 80.8, 80.1, 75.0, 72.3, 69.5, 68.2. Anal. calcd for C_38_H_40_N_4_O_10_: C, 64.04; H, 5.66; N, 7.86. Found: C, 63.80; H, 5.60; N, 7.50.

#### 3.5.7. Physicochemical and spectral data for 4,6-*O*-butylidene-*N*-(2-acetamido-2′′-amino-*ortho*-bisazobenzyl)-β-d-glucopyranosyl amine, 13

Compound, 13 was obtained by the reaction of 2-acetamido-2′′-amino-*ortho*-bisazobenzene 12 (1.2 mmol, 0.91 g) and 4,6-*O*-butylidene-β-d-glucopyranose 1a (1.0 mmol, 0.5 g) as a red solid. Yield: 0.80 g (66%); mp 156–158 °C; ^1^H NMR (CDCl_3_ + DMSO-d_6_, 300 MHz), *δ* 8.63 (d, *J* = 8.4 Hz, 1H, Ar-*H*), 7.79–7.85 (m, 2H, Ar-H), 7.72 (t, *J* = 5.7 Hz, 1H, Ar-H), 7.53–7.59 (m, 2H, Ar-H), 7.40–7.47 (m, 2H, Ar-H), 7.19 (d, *J* = 5.1 Hz, 3H, Ar-H), 6.80 (t, *J* = 6.0 Hz, 2H, Ar-H), 5.87 (s, 1H, Ace-H), 4.54 (t, *J* = 5.1 Hz, 1H, Ano-H), 4.05–4.10 (q, *J* = 4.8 Hz, 1H, –NH), 3.84–3.90 (m, 3H, Sac-H), 3.47–3.51 (m, 2H, Sac-OH), 3.20–3.33 (m, 2H, Sac-H), 2.70 (s, 1H, Sac-H), 2.02 (s, 3H, –NHCOCH_3_), 1.62 (t, *J* = 4.5 Hz, 2H, Sac-H), 1.39–1.46 (q, *J* = 7.2 Hz, *J* = 7.5 Hz, 2H, Sac-H), 0.91 (t, *J* = 7.5 Hz, 3H, Sac-CH_3_). ^13^C NMR (CDCl_3_ + DMSO-d_6_, 75 MHz) *δ* 168.6, 147.2, 147.1, 142.1, 139.3, 137.1, 135.7, 132.5 (2C), 132.3, 131.1, 130.3, 129.6, 123.0, 120.1, 118.0, 116.8, 116.7, 116.4, 101.9, 97.0, 80.4, 73.0, 70.6, 68.3, 61.9, 35.9, 24.6, 17.0, 13.5. Anal. calcd for C_30_H_34_N_6_O_6_: C, 62.71; H, 5.96; N, 14.63. Found: C, 61.90; H, 5.80; N, 14.50.

#### 3.5.8. Physicochemical and spectral data for 4,6-*O*-ethylidene-*N*-(2-acetamido-2′′-amino-*ortho*-bisazobenzyl)-β-d-glucopyranosyl amine, 14

Compound, 14 was obtained by the reaction of 2-acetamido-2′′-amino-*ortho*-bisazobenzene, 12 (0.52 mmol, 1.04 g) and 4,6-*O*-ethylidene-β-d-glucopyranose 1b (0.5 mmol, 0.5 g) as a red solid. Yield: 1.00 g (76%); mp 152–154 °C; ^1^H NMR (CDCl_3_ + DMSO-d_6_, 300 MHz), *δ* 8.65 (d, *J* = 8.1 Hz, 2H, Ar-H), 7.76–7.93 (m, 3H, Ar-H), 7.60 (t, *J* = 7.5 Hz, 1H, Ar-H), 7.54 (t, *J* = 6.3 Hz, 1H, Ar-H), 7.21–7.35 (m, 2H, Ar-H), 6.97 (d, *J* = 8.7 Hz, 1H, Ar-H), 6.71–6.91 (m, 2H, Ar-H), 6.16 (s, 1H, –NH), 5.16 (s, 1H, Ace-H), 4.71–4.76 (q, *J* = 4.8 Hz, 1H, Ano-H), 4.41 (s, 1H, –NH), 4.61 (s, 3H, Sac-H), 4.02–4.12 (m, 1H, Sac-H), 3.83–3.89 (q, *J* = 4.2 Hz, 2H, Sac-OH), 3.46–3.54 (m, 1H, Sac-H), 3.21–3.32 (m, 1H, Sac-H), 1.88 (s, 3H, –NHCOCH_3_), 1.37 (t, *J* = 4.8 Hz, 3H, Sac-CH_3_). ^13^C NMR (CDCl_3_ + DMSO-d_6_, 75 MHz) *δ* 174.2, 152.3, 147.7, 142.6, 140.3, 138.4, 136.3 (2C), 134.8, 132.4 (2C), 128.0 (2C), 125.5, 124.0, 123.2, 118.5 (2C), 104.1, 102.2, 97.8, 85.6, 80.8, 78.2, 73.3, 66.8, 29.5, 25.1. Anal. calcd for C_28_H_30_N_6_O_6_: C, 61.53; H, 5.53; N, 15.38. Found: C, 61.20; H, 5.40; N, 15.20.

#### 3.5.9. Physicochemical and spectral data for 4,6-*O*-benzylidene-*N*-(2-acetamido-2′′-amino-*ortho*-bisazobenzyl)-β-d-glucopyranosyl amine, 15

Compound, 15 was obtained by the reaction of 2-acetamido-2′′-amino-*ortho*-bisazobenzene, 12 (0.52 mmol, 0.54 g) and 4,6-*O*-ethylidene-β-d-glucopyranose 1c (0.5 mmol, 0.5 g) as a red solid. Yield: 0.85 g (85%); mp 153–155 °C; ^1^H NMR (CDCl_3_ + DMSO-d_6_, 300 MHz), *δ* 8.52 (d, *J* = 8.1 Hz, 1H, Ar-H), 8.10 (t, 3H, Ar-H), 7.73 (d, *J* = 5.4 Hz, 1H, Ar-H), 7.65 (d, *J* = 7.5 Hz, 2H, Ar-H), 7.40–7.51 (m, 3H, Ar-H), 7.25 (d, *J* = 3.3 Hz, 3H, Ar-H), 7.10 (t, *J* = 7.5 Hz, 2H, Ar-H), 6.69–6.76 (m, 4H, Ar-H, –NH), 6.10 (s, 1H, Ar-H), 5.43 (s, 1H, Ace-H), 5.12 (s, 1H, –NH), 4.59 (s, 1H, Ano-H), 4.41 (d, 1H, Sac-H), 4.11–4.20 (m, 1H, –NH), 3.77–3.97 (m, 1H, Sac-H), 3.66 (d, *J* = 6.6 Hz, 2H, Sac-H), 3.37–3.67 (m, 2H, Sac-OH), 1.95 (s, 3H, –NHCOCH_3_). ^13^C NMR (CDCl_3_ + DMSO-d_6_, 75 MHz) *δ* 168.9, 147.5 (2C), 142.5, 139.6, 137.3, 136.0 (2C), 132.7, 132.5 (2C), 131.4, 130.7, 129.9, 128.8, 127.9, 126.3, 123.3 (2C), 120.5, 118.1, 117.1, 116.9 (2C), 116.5, 101.6, 97.4, 93.0, 81.4, 73.2, 68.6, 62.0, 24.8. Anal. calcd for C_33_H_32_N_6_O_6_: C, 65.12; H, 5.30; N, 13.81. Found: C, 64.90; H, 5.20; N, 13.60.

## 4. Conclusion

By introducing 4,6-*O*-protected glucose core into azobenzene moiety, we obtained a versatile gelator which can widely gel varieties of organic solvents. The electron microscopy and spectral studies showed that the gel formation is due to aggregation of molecules into aggregated bundles or helical fibres through cooperative interactions of hydrogen bonding of sugar moiety, π–π interactions of azobenzene groups, and hydrophobicity of alkyl chains. From this we have obtained new insight toward the molecular design in more effective organogelator for organic solvents. Despite from the wide study of azobenzenes this class of molecules shows exciting activities. The biological evaluation of the compounds are under progress and we are planing to reveal those informations in our upcoming research paper.

## Conflicts of interest

There are no conflicts to declare.

## Supplementary Material

RA-009-C9RA08033C-s001
