# Supplementary material for: Insights into a novel class of azobenzenes incorporating 4,6-O-protected sugars as photo-responsive organogelators
Source: RSC Adv. 2019 Dec 19;9(72):42219–27. doi: 10.1039/c9ra08033c (PMC9076560; doi:10.1039/c9ra08033c)

Electronic Supplementary Material (ESI) for RSC Advances.
This journal is © The Royal Society of Chemistry 2019

Insights into a novel class of azobenzene incorporated 4,6-*O*-protected sugar as photo-responsive organogelators

***Bhavya P. V*[a]*., Vasanthan RabeccaJenifer*[a]*, Panneerselvam Muthuvel*[b]*, Thangamuthu Mohan Das*[a,b,*]**

[a] *Department of Chemistry, School of Basic and Applied sciences, Central University of Tamil Nadu (CUTN), Thiruvarur 610 005, INDIA*

[b] *Department of Organic Chemistry, University of Madras, Guindy Campus, Chennai - 600 025, INDIA*

---

* Corresponding author. Tel.: +91 9965048959

E-mail addresses: tmohandas@cutn.ac.in, tmdas_72@yahoo.com (T. Mohan Das).

Supporting information

Figure S.1. HRMS spectrum of compound, **4**	S3
Figure S.2. HRMS spectrum of compound, **5**	S4
Figure S.3. HRMS spectrum of compound, **6**	S5
Figure S.4. HRMS spectrum of compound, **8**	S6
Figure S.5. HRMS spectrum of compound, **9**	S7
Figure S.6. HRMS spectrum of compound, **10**	S8
Figure S.7. HRMS spectrum of compound, **12**	S9
Figure S.8. 1H NMR spectrum of compound, **5**	S10
Figure S.9. 13C NMR spectrum of compound, **5**	S11
Figure S.10. 1H NMR spectrum of compound, **6**	S12
Figure S.11. 13C NMR spectrum of compound, **6**	S13
Figure S.12. 1H NMR spectrum of compound, **7**	S14
Figure S.13. 13C NMR spectrum of compound, **7**	S15
Figure S.14. 1H NMR spectrum of compound, **9**	S16
Figure S.15. 13C NMR spectrum of compound, **9**	S17
Figure S.16. 1H NMR spectrum of compound, **10**	S18
Figure S.17. 13C NMR spectrum of compound, **10**	S19
Figure S.18. 1H NMR spectrum of compound, **11**	S20
Figure S.19. 13C NMR spectrum of compound, **11**	S21
Figure S.20. 1H NMR spectrum of compound, **13**	S22
Figure S.21. 13C NMR spectrum of compound, **13**	S23
Figure S.22. 1H NMR spectrum of compound, **14**	S24
Figure S.23. 13C NMR spectrum of compound, **14**	S25
Figure S.24. 1H NMR spectrum of compound, **15**	S26
Figure S.25. 13C NMR spectrum of compound, **15**	S27

Figure S1. HRMS spectrum of compound 4

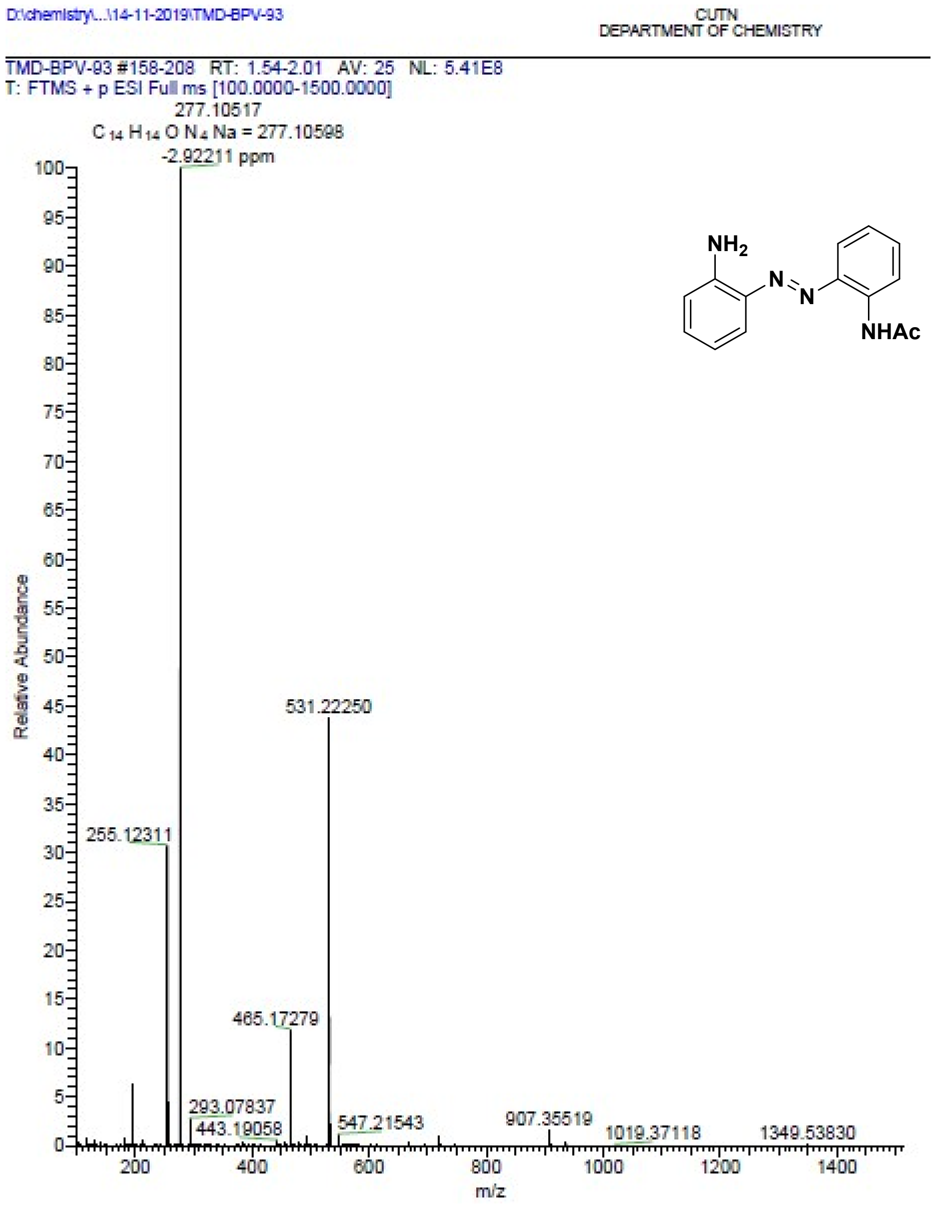

Figure S2. HRMS spectrum of compound, 5

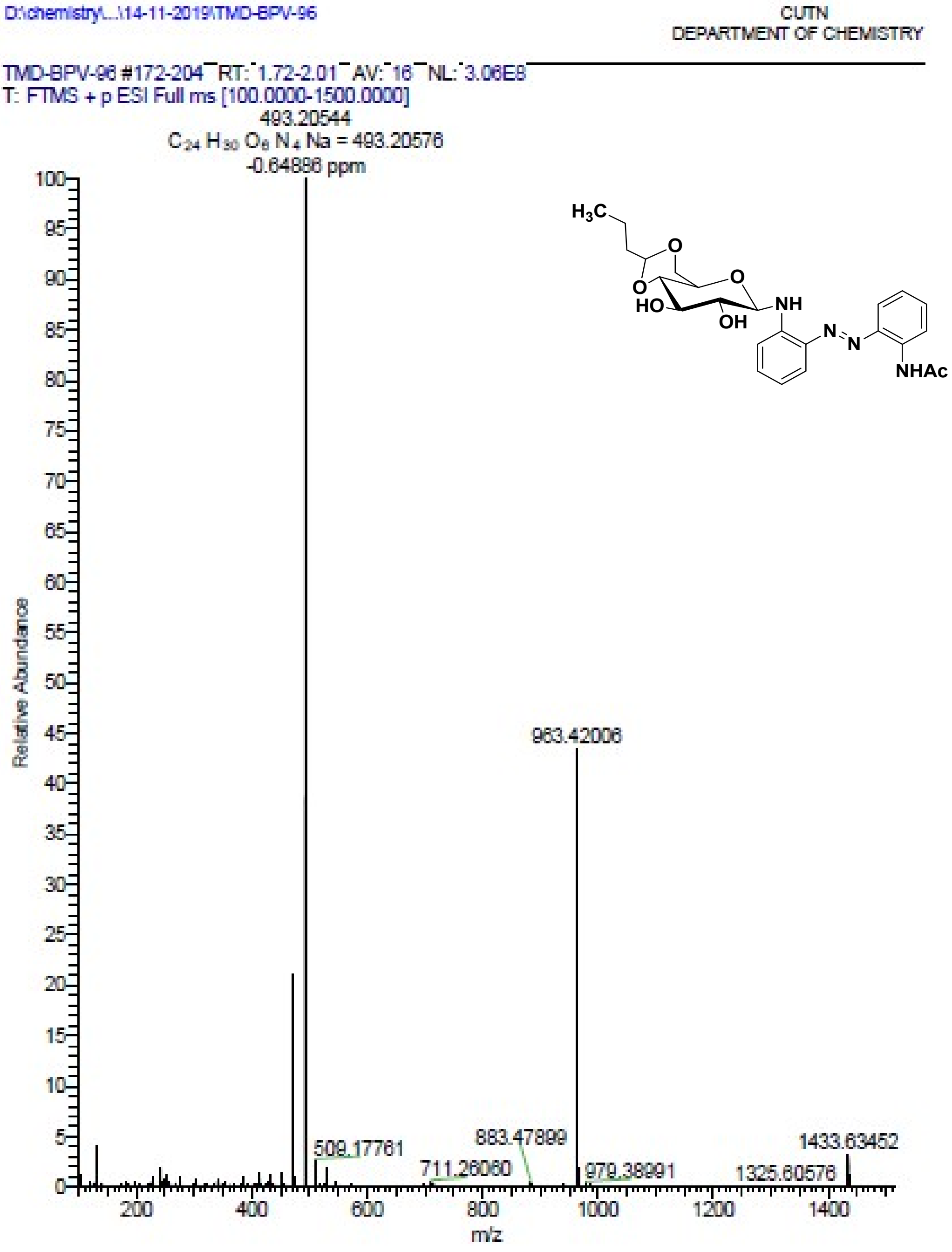

Figure S3. HRMS spectrum of compound, 6

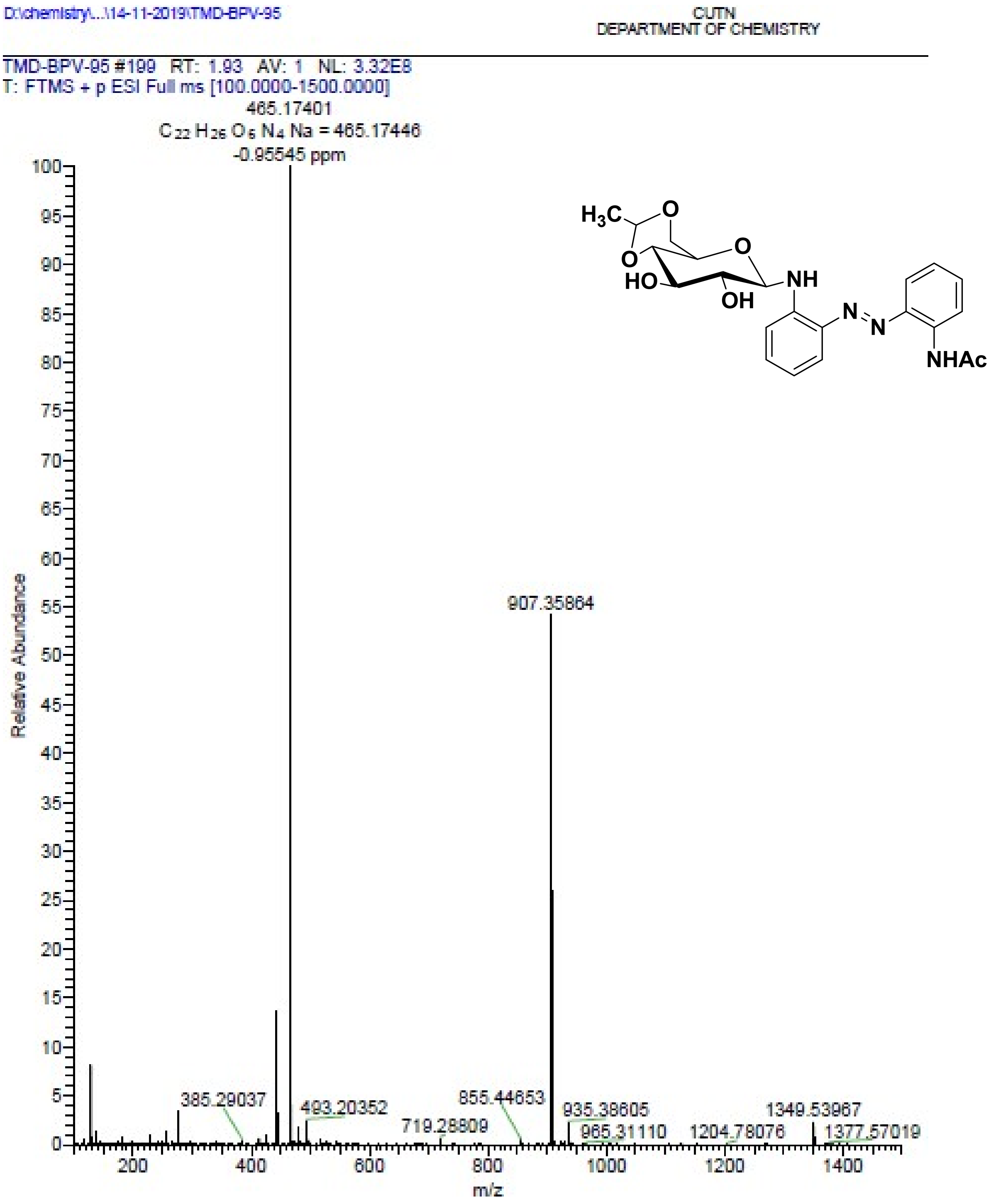

Figure S4. HRMS spectrum of compound 8

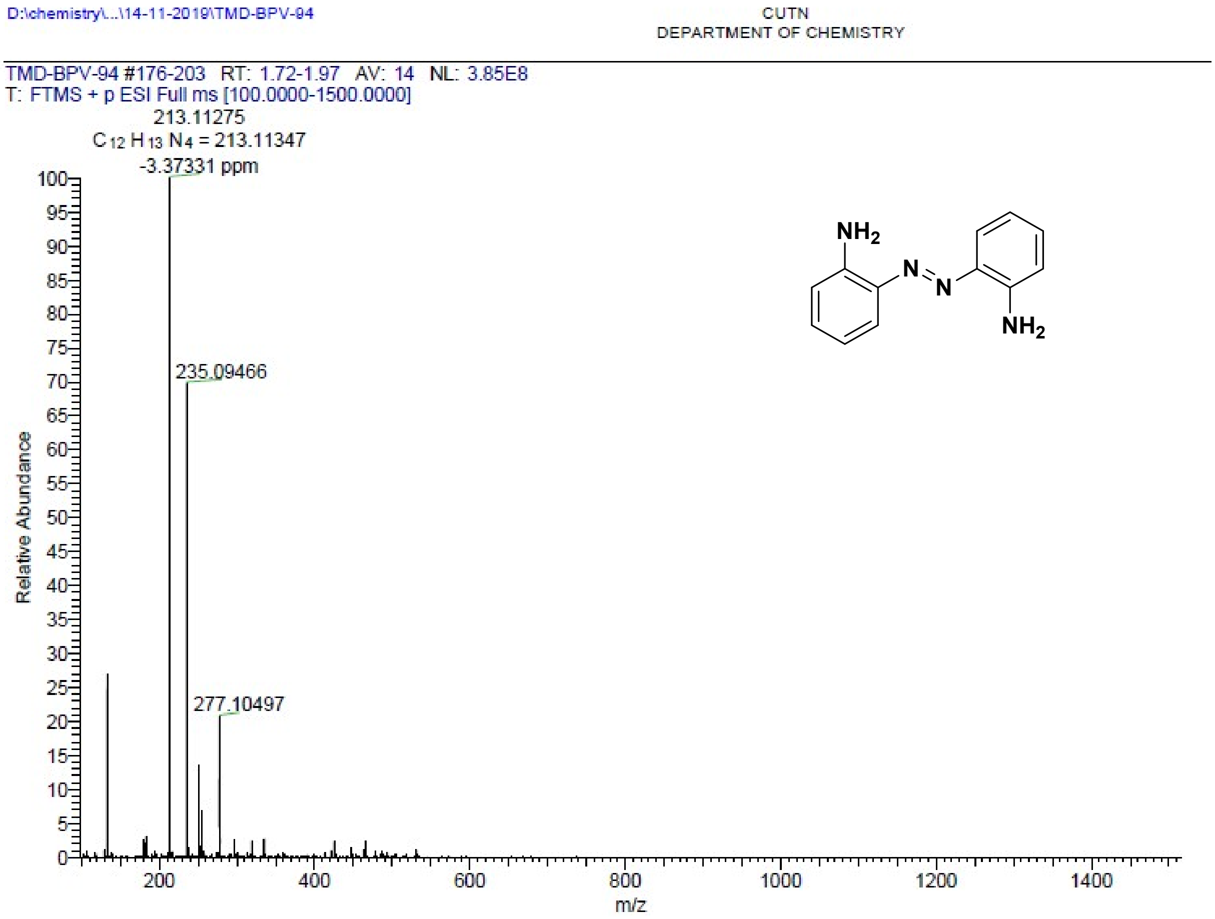

Figure S5. HRMS spectrum of compound,9

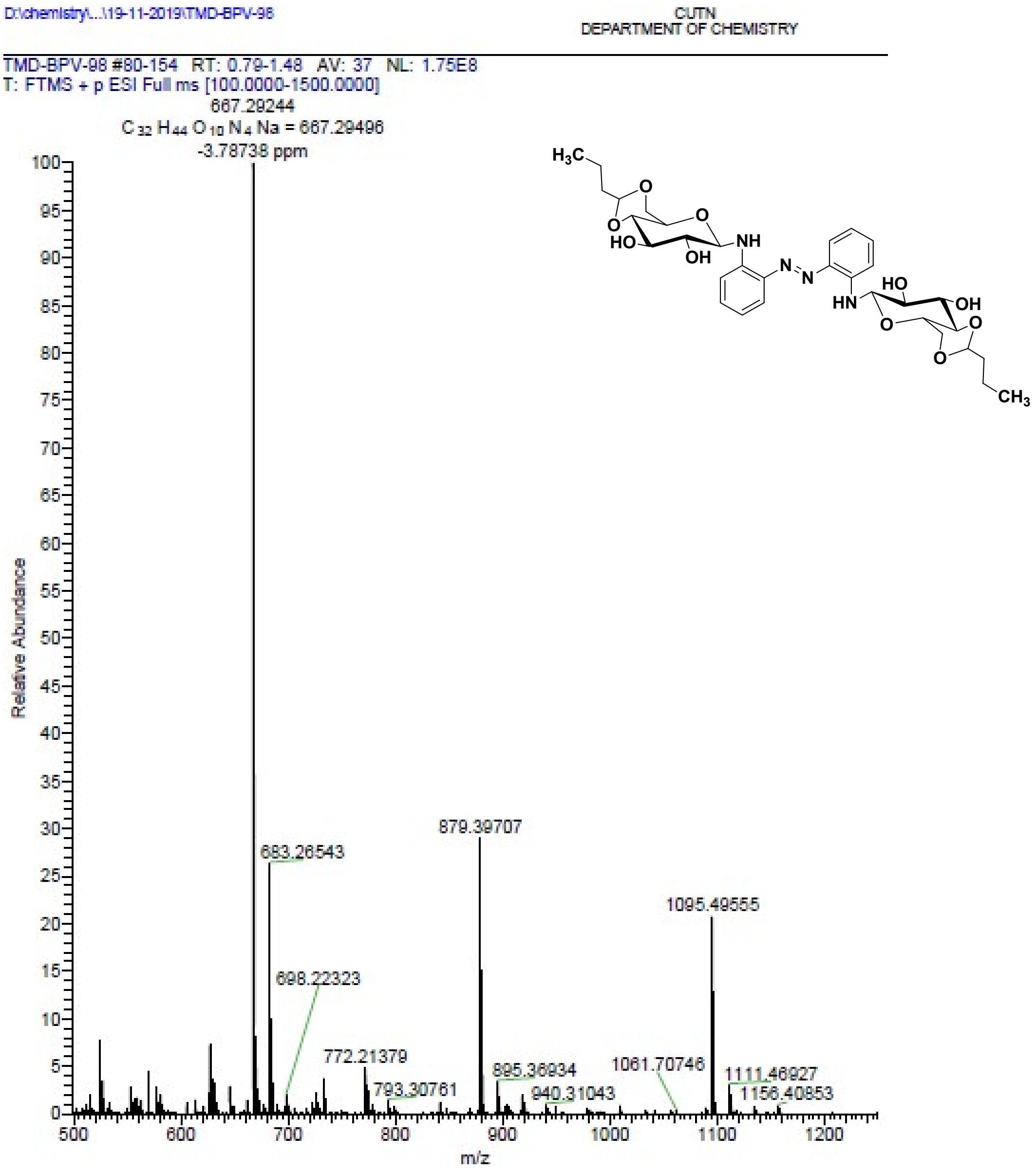

Figure S6. HRMS spectrum of compound,10

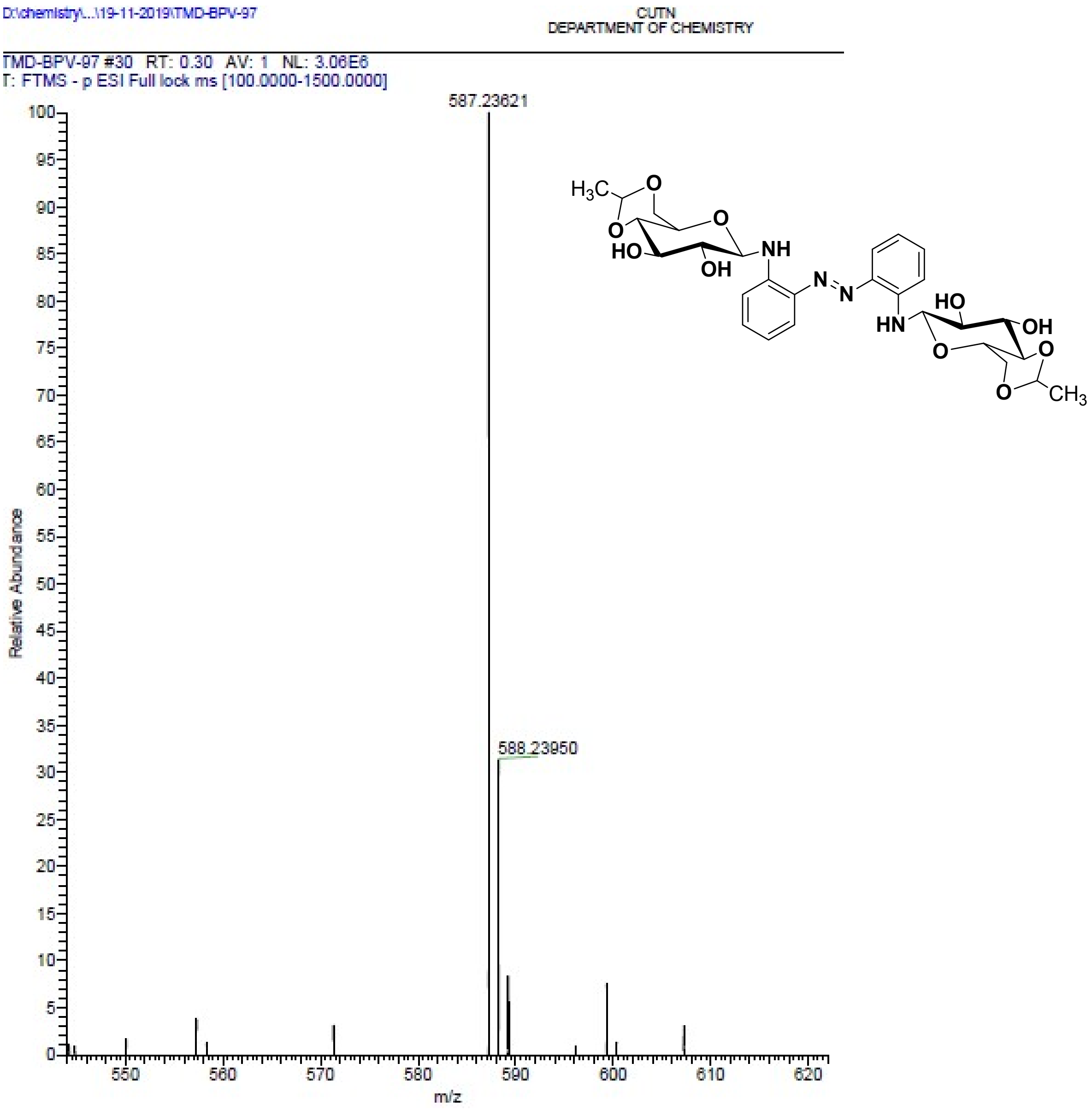

Figure S7. HRMS spectrum of compound, 12

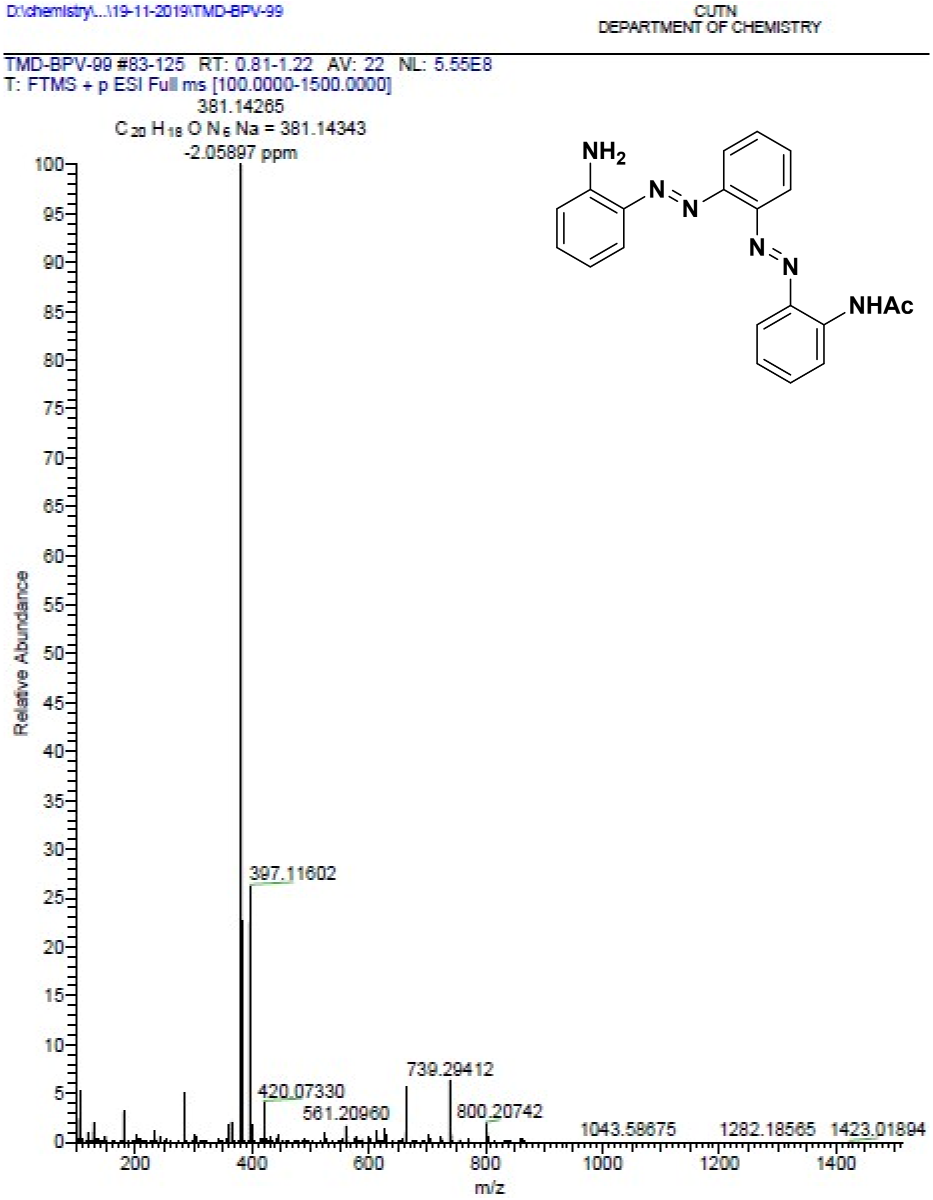

Figure S8. ^{1}H NMR Spectrum of compound, 5 ($CDCl_3$ (0.6mL)+DMSO-d_6 (0.1mL), 300MHz)

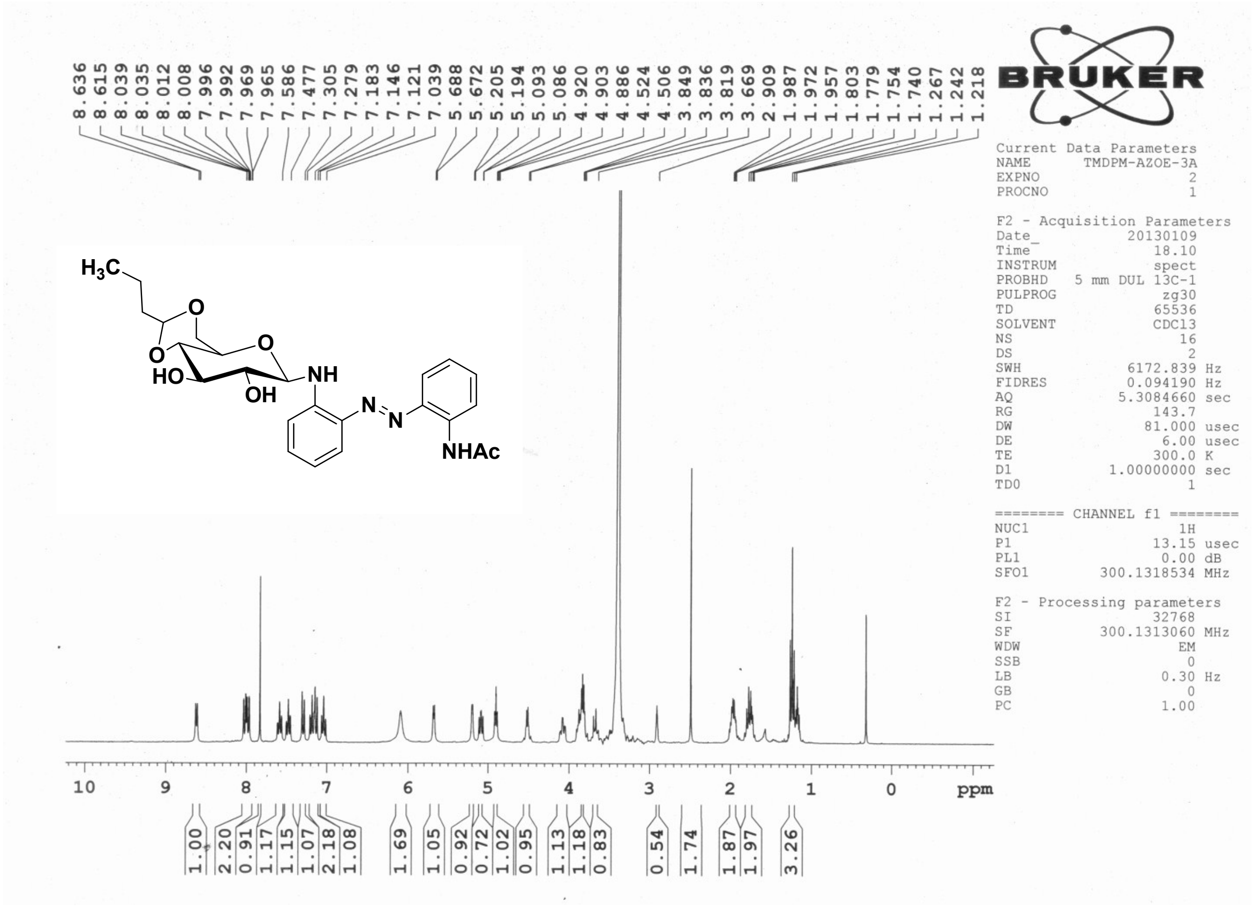

Figure S9. ^{13}C NMR Spectrum of compound, 5 ($CDCl_3$(0.6mL) +DMSO- d_6 (0.1mL), 75MHz)

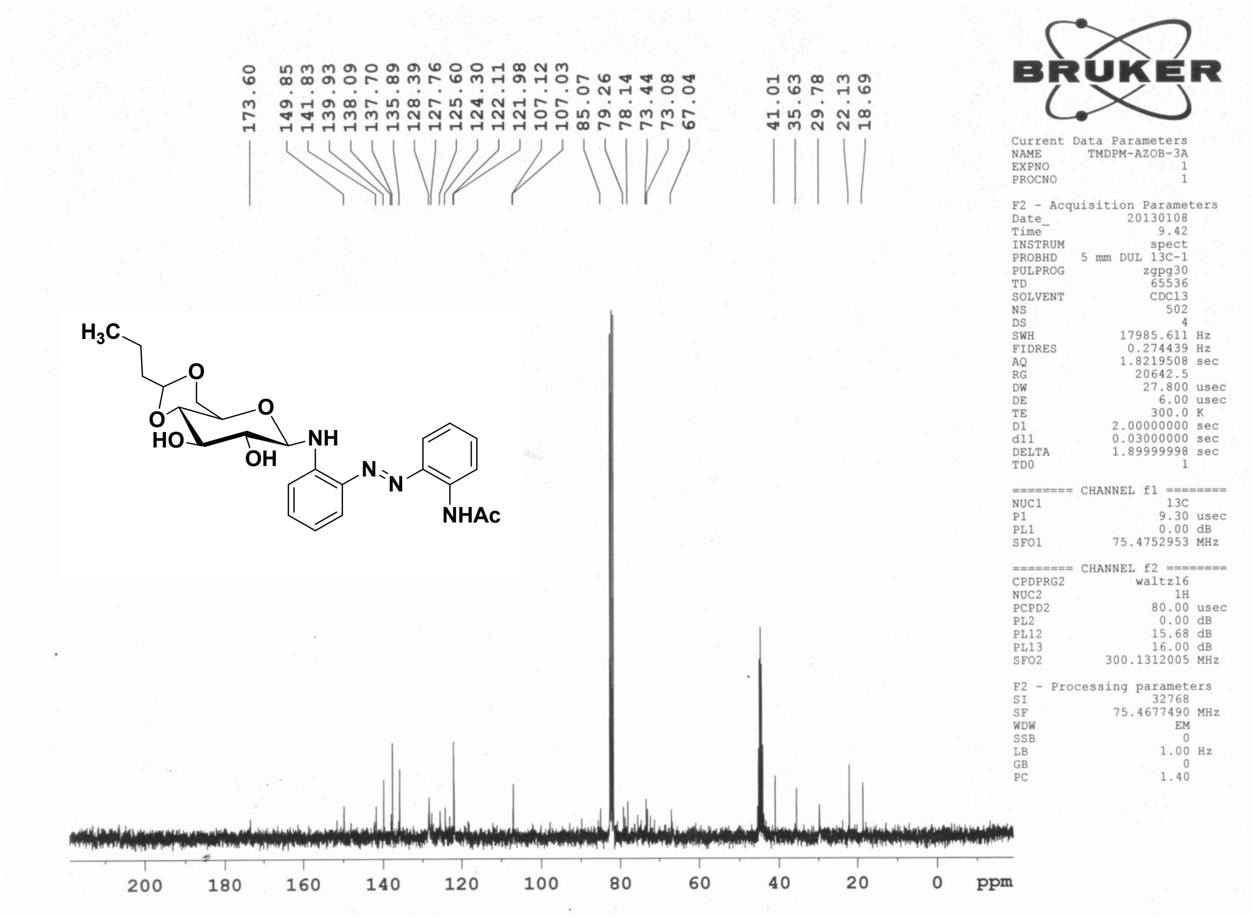

Figure S10. ^{1}H NMR Spectrum of compound, 6 ($CDCl_3$(0.6mL) +DMSO- d_6 (0.1mL), 300MHz)

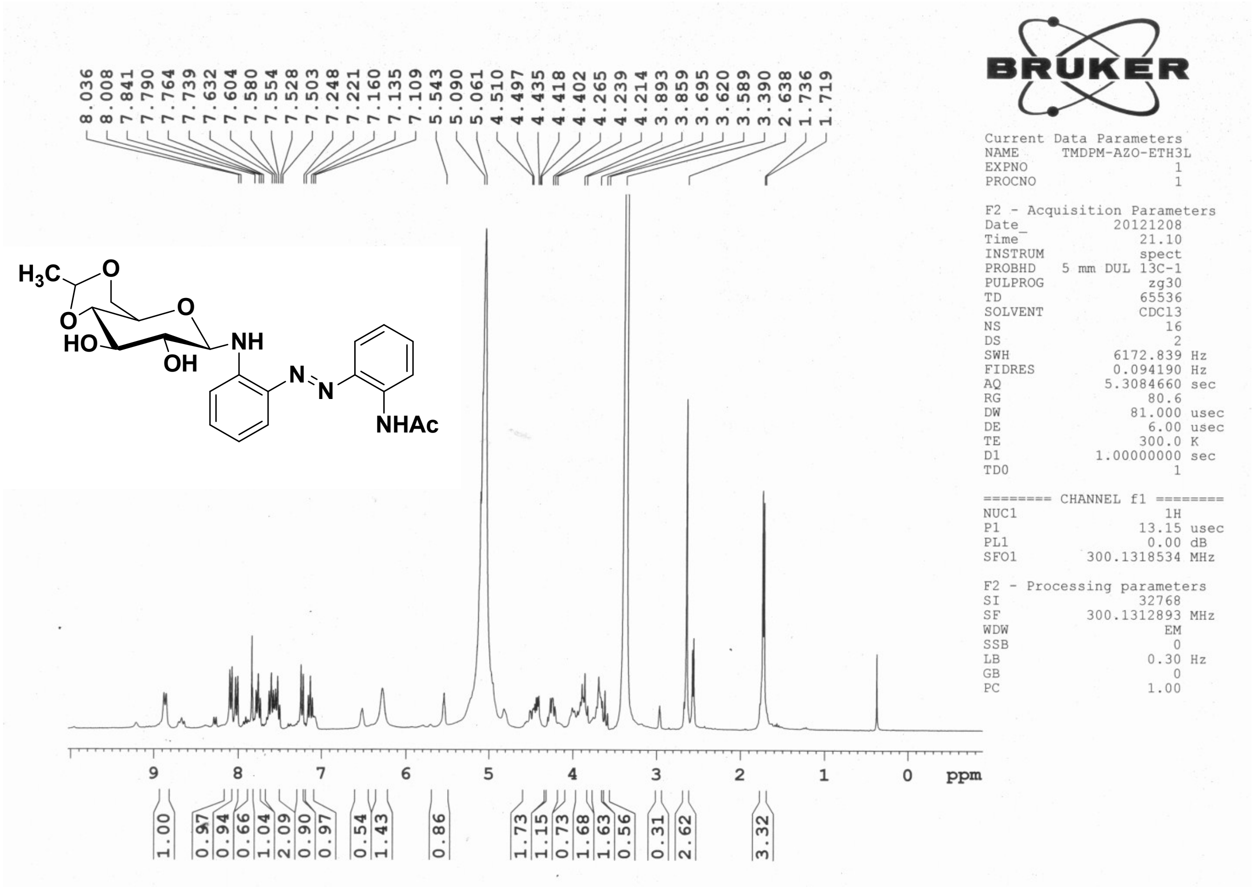

Figure S11. ^{13}C NMR Spectrum of compound 6 ($CDCl_3$(0.6mL) +DMSO- d_6 (0.1mL), 75MHz)

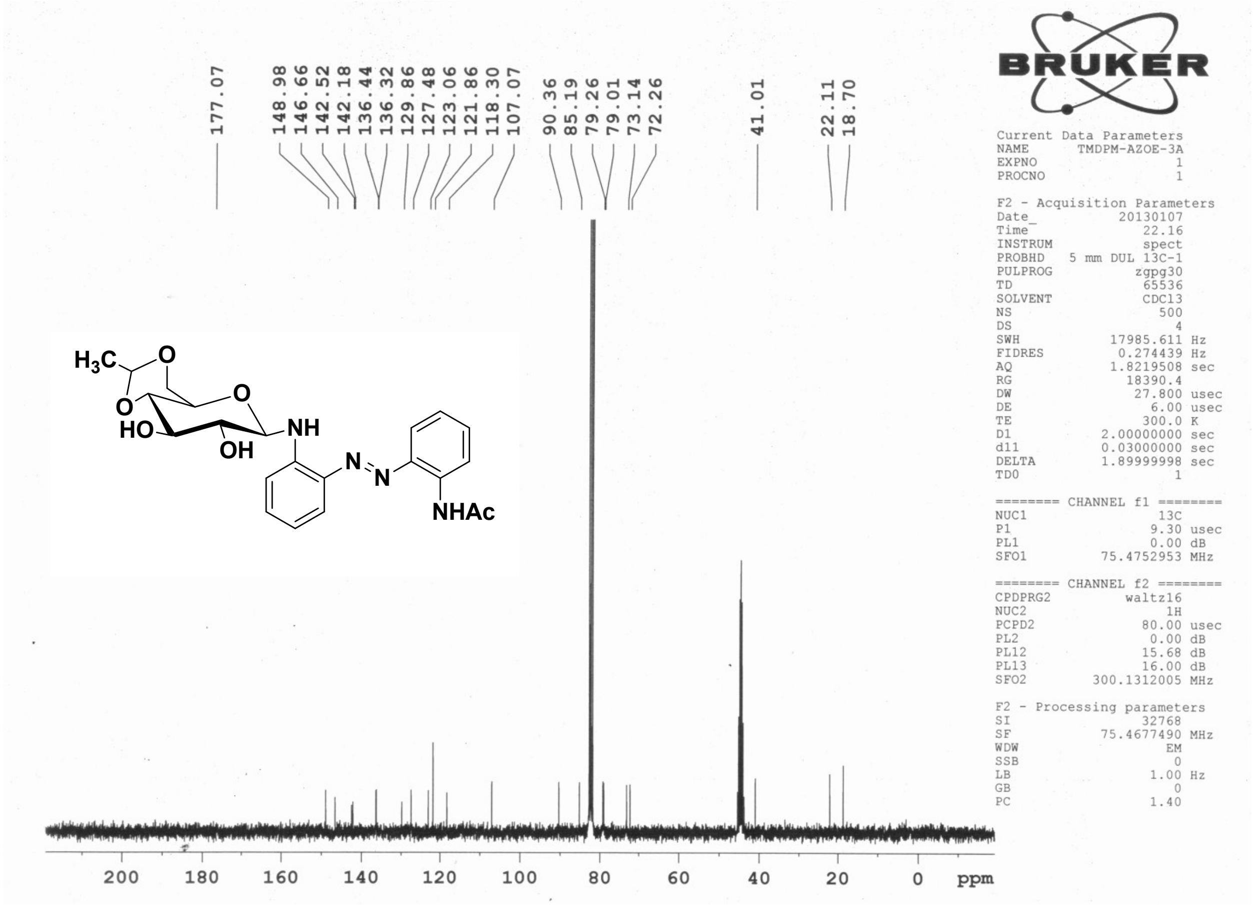

Figure S12. ^{1}H NMR Spectrum of compound, 7 ($CDCl_3$(0.6mL) +DMSO- d_6 (0.1mL), 300MHz)

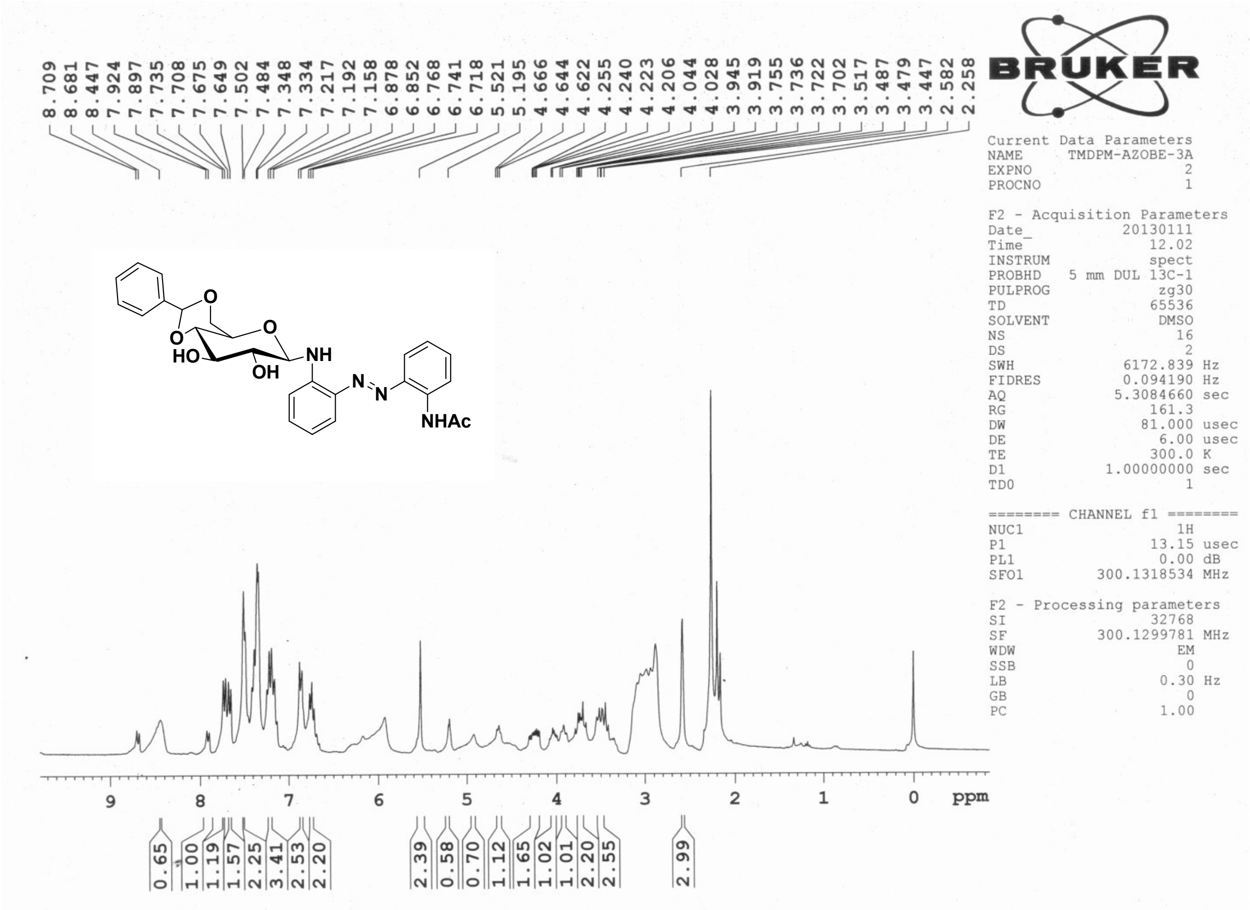

Figure S13. ^{13}C NMR Spectrum of compound 7 ($CDCl_3$(0.6mL) +DMSO-d_6 (0.1mL), 75MHz)

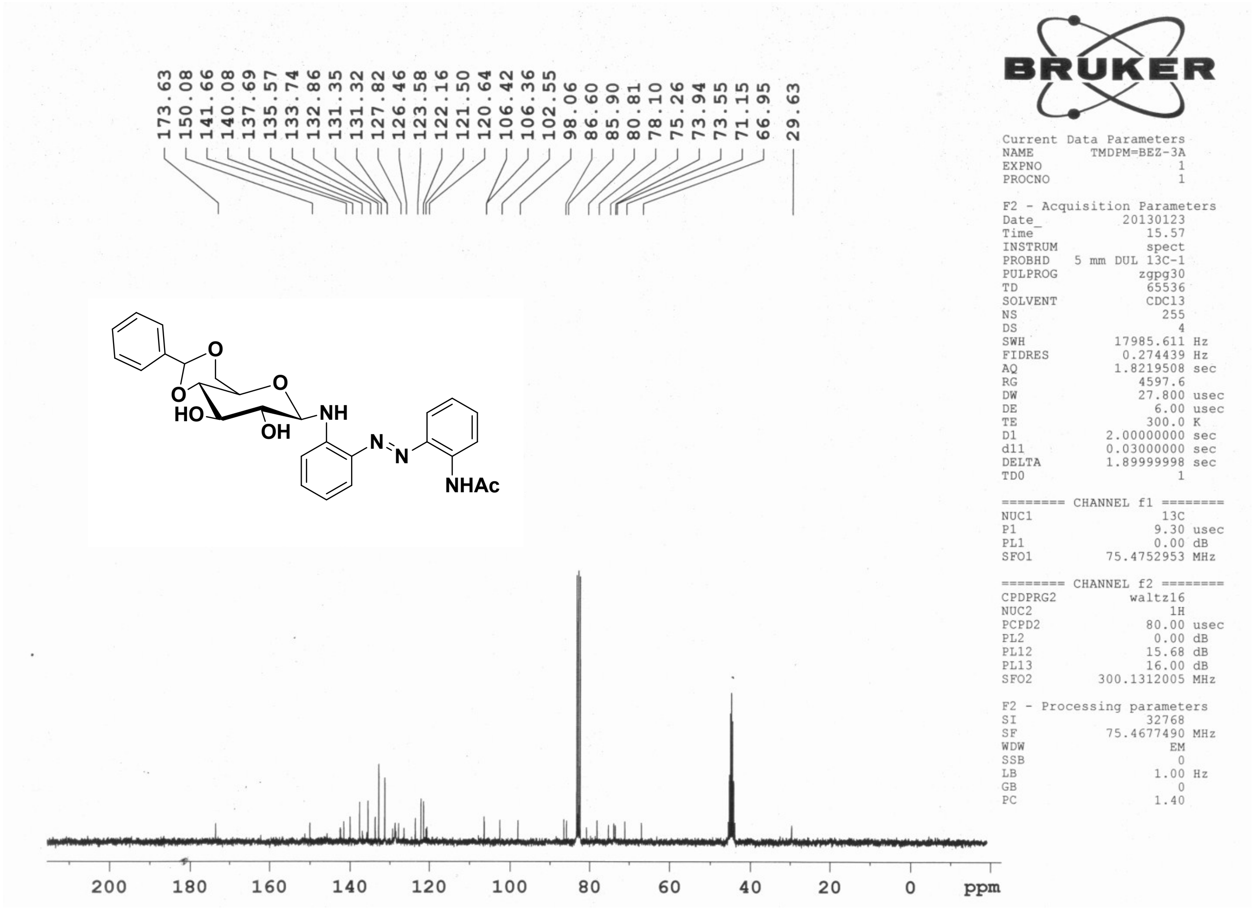

Figure S14. ^1H NMR Spectrum of compound, 9 ($CDCl_3$(0.6mL) +DMSO- d_6 (0.1mL), 300MHz)

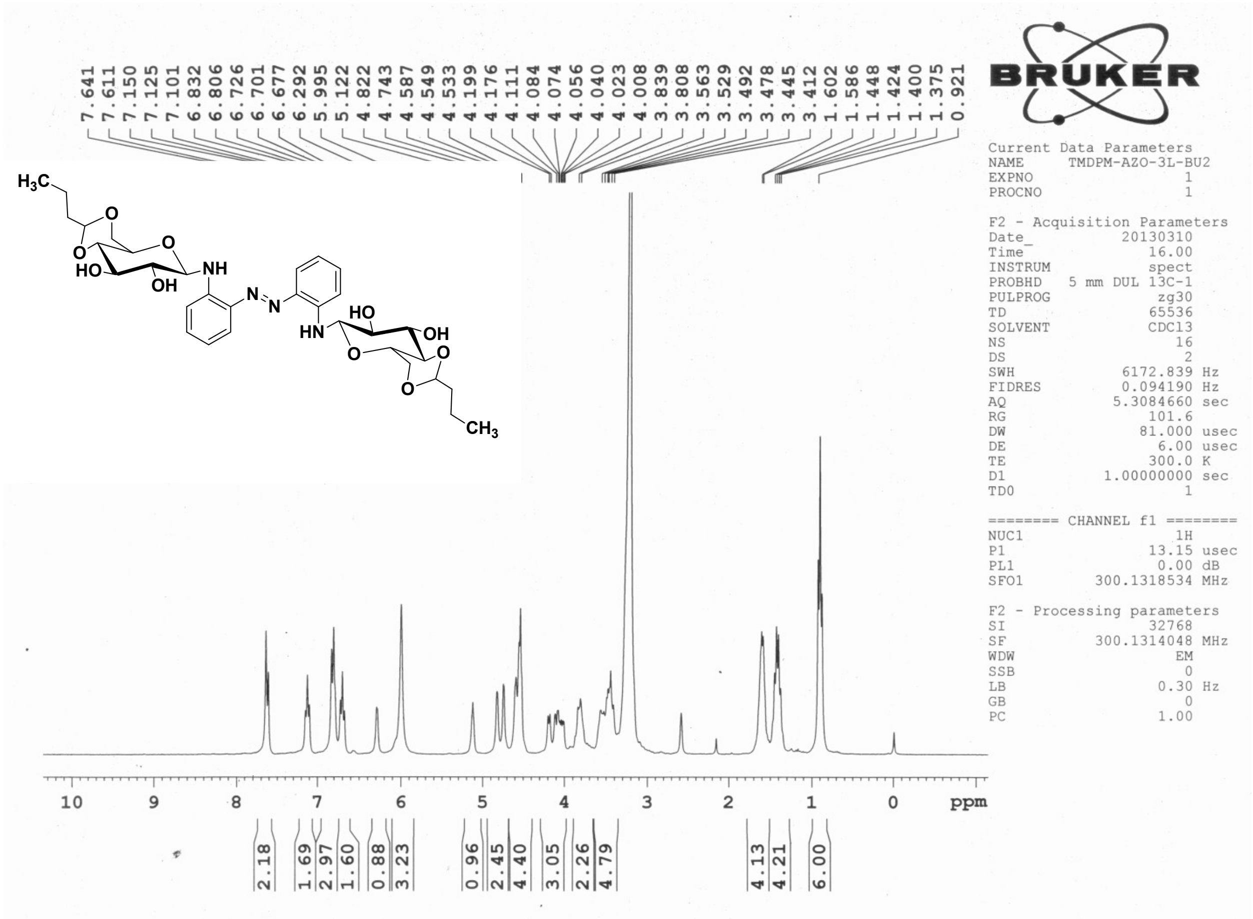

Figure S15. ^{13}C NMR Spectrum of compound 9 ($CDCl_3$(0.6mL) +DMSO- d_6 (0.1mL), 75MHz)

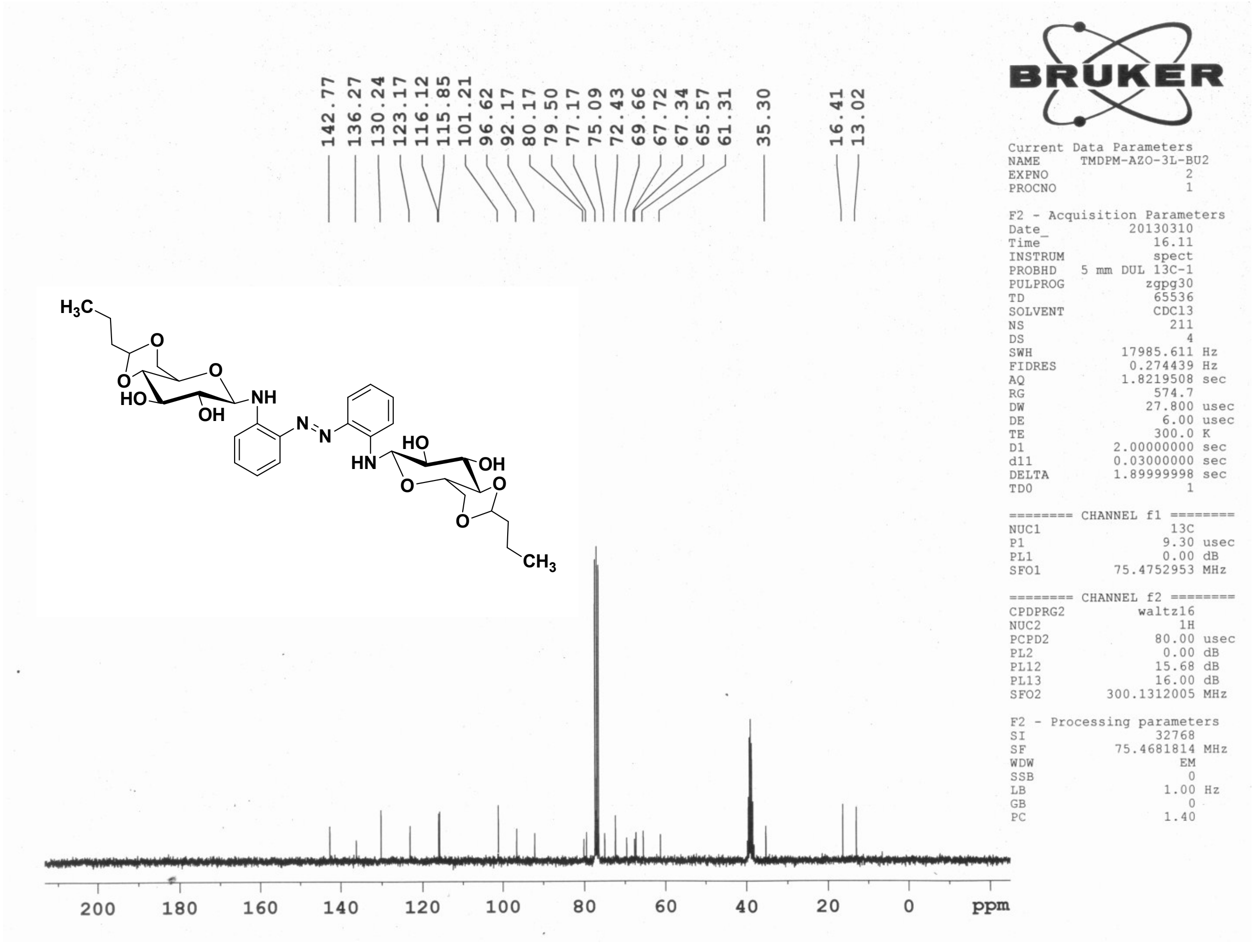

Figure S16. ^{1}H NMR Spectrum of compound, 10 ($CDCl_3$(0.6mL) +DMSO- d_6 (0.1mL), 300MHz)

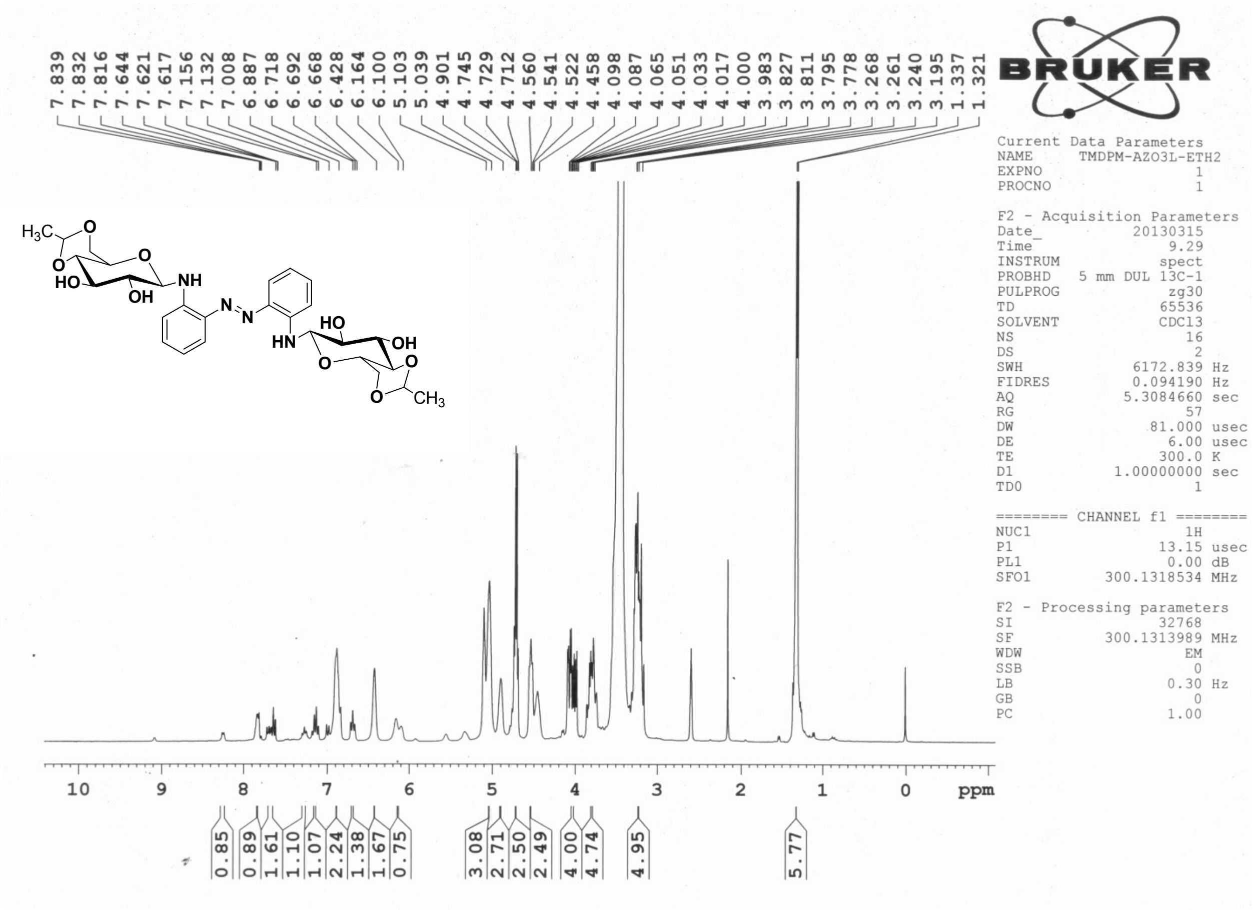

Figure S17. ^{13}C NMR Spectrum of compound, 10 ($CDCl_3$(0.6mL) +DMSO- d_6 (0.1mL), 75MHz)

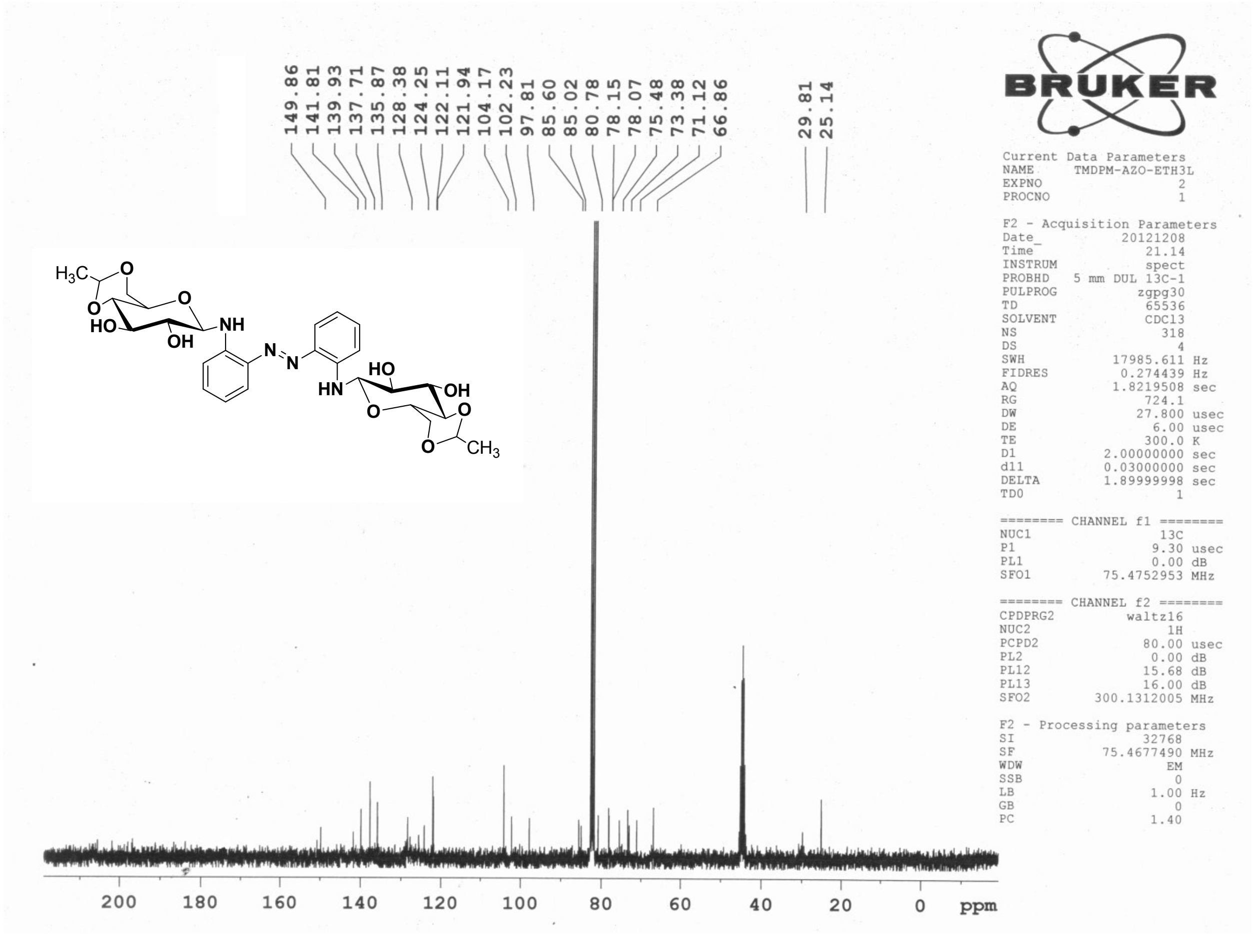

Figure S18. ^{1}H NMR Spectrum of compound, 11 ($CDCl_3$(0.6mL) +DMSO- d_6 (0.1mL), 300MHz)

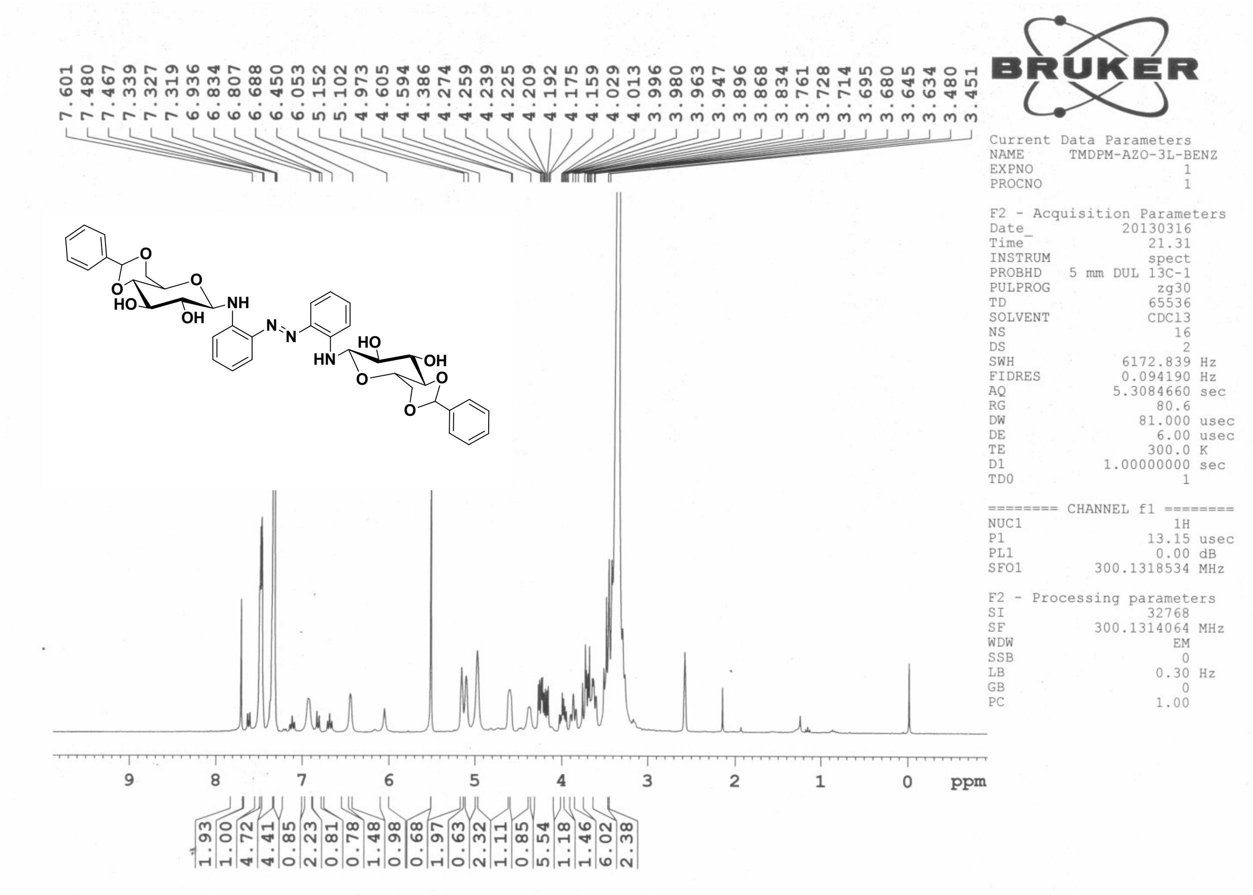

Figure S19. ^{13}C NMR Spectrum of compound 11 ($CDCl_3$(0.6mL) +DMSO- d_6 (0.1mL), 75MHz)

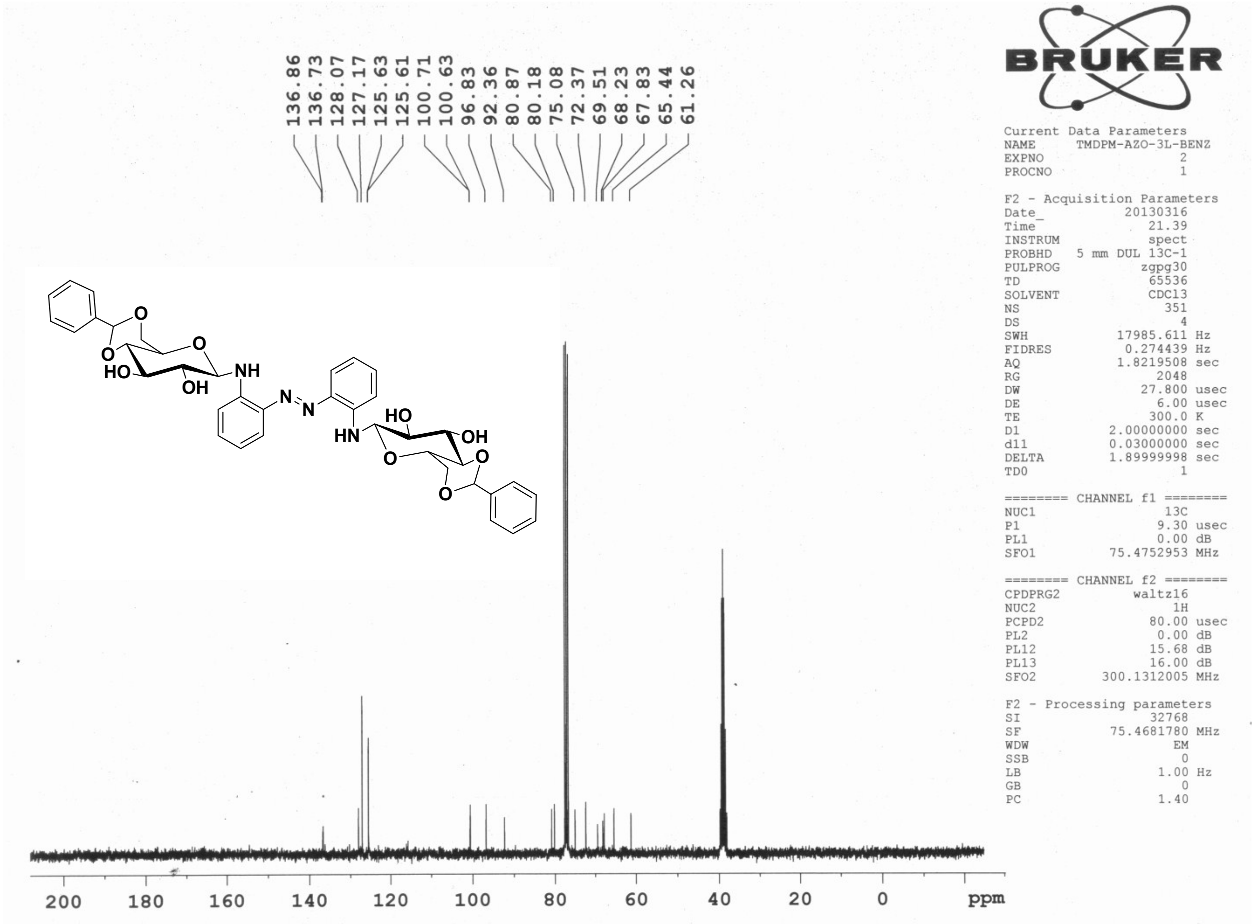

Figure S20. ^{1}H NMR Spectrum of compound, 13 ($CDCl_3$(0.6mL) +DMSO- d_6 (0.1mL), 300MHz)

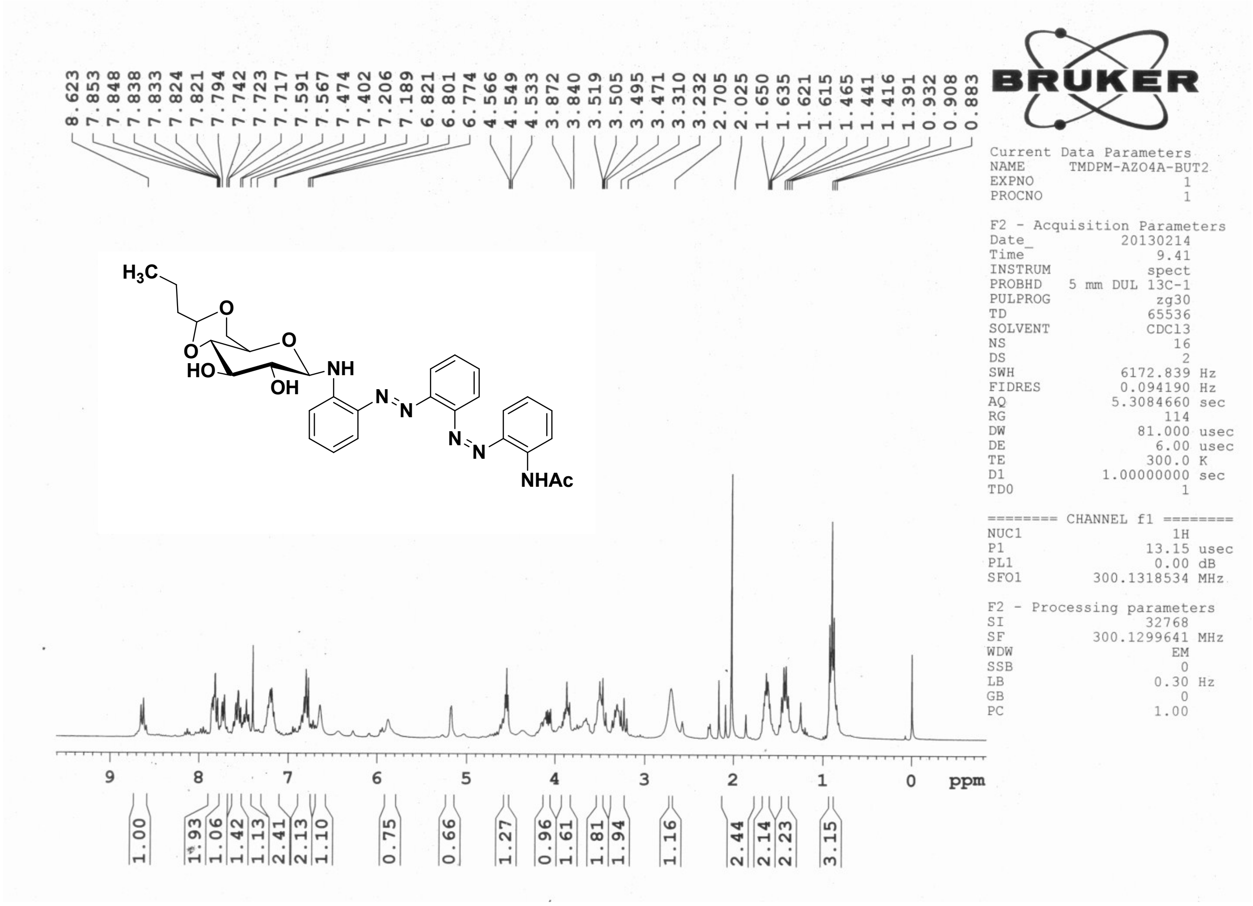

Figure S21. ^{13}C NMR Spectrum of compound 13 ($CDCl_3$(0.6mL) +DMSO- d_6 (0.1mL), 75MHz)

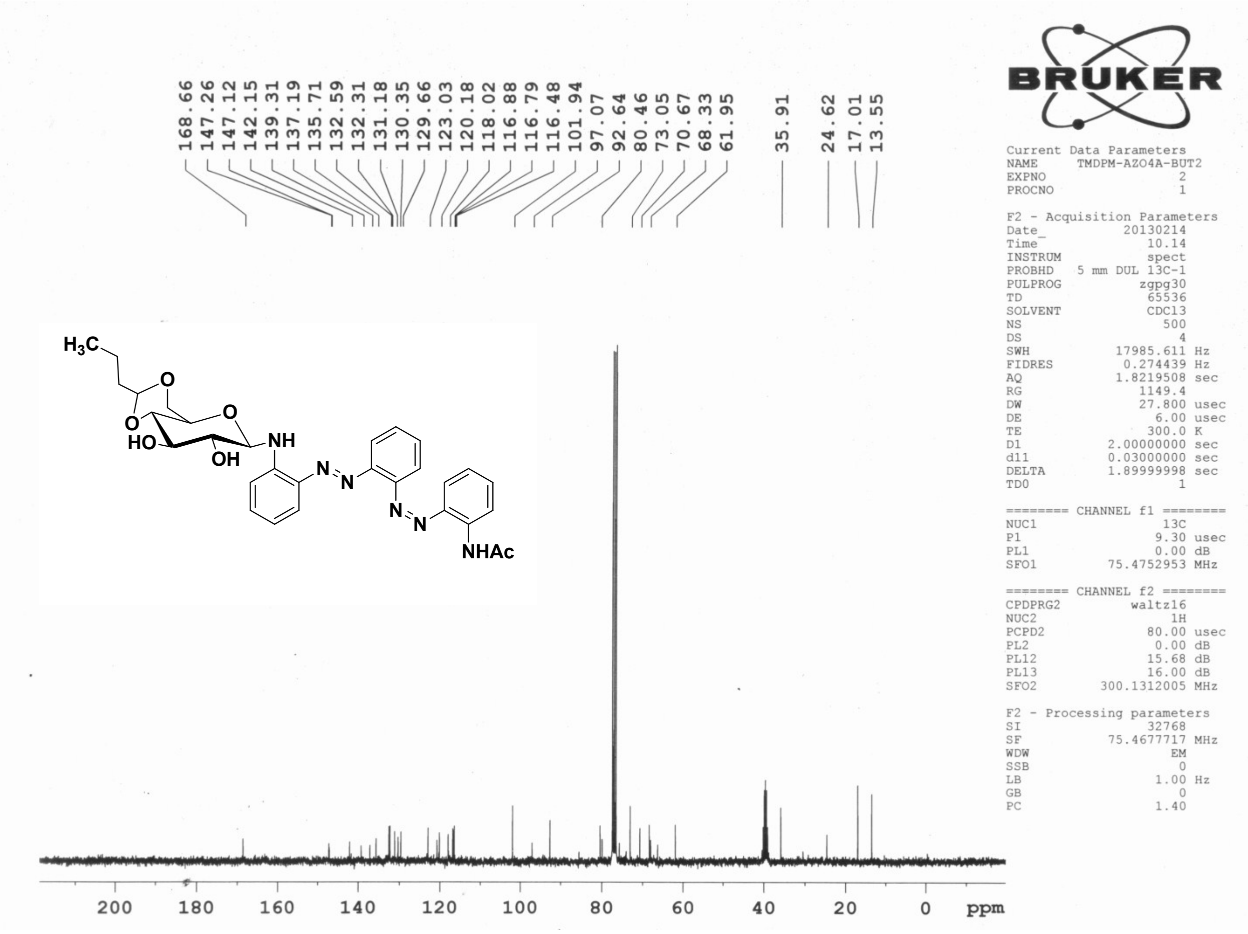

Figure S22. ^{1}H NMR Spectrum of compound 14 ($CDCl_3$(0.6mL) +DMSO- d_6 (0.1mL), 300MHz)

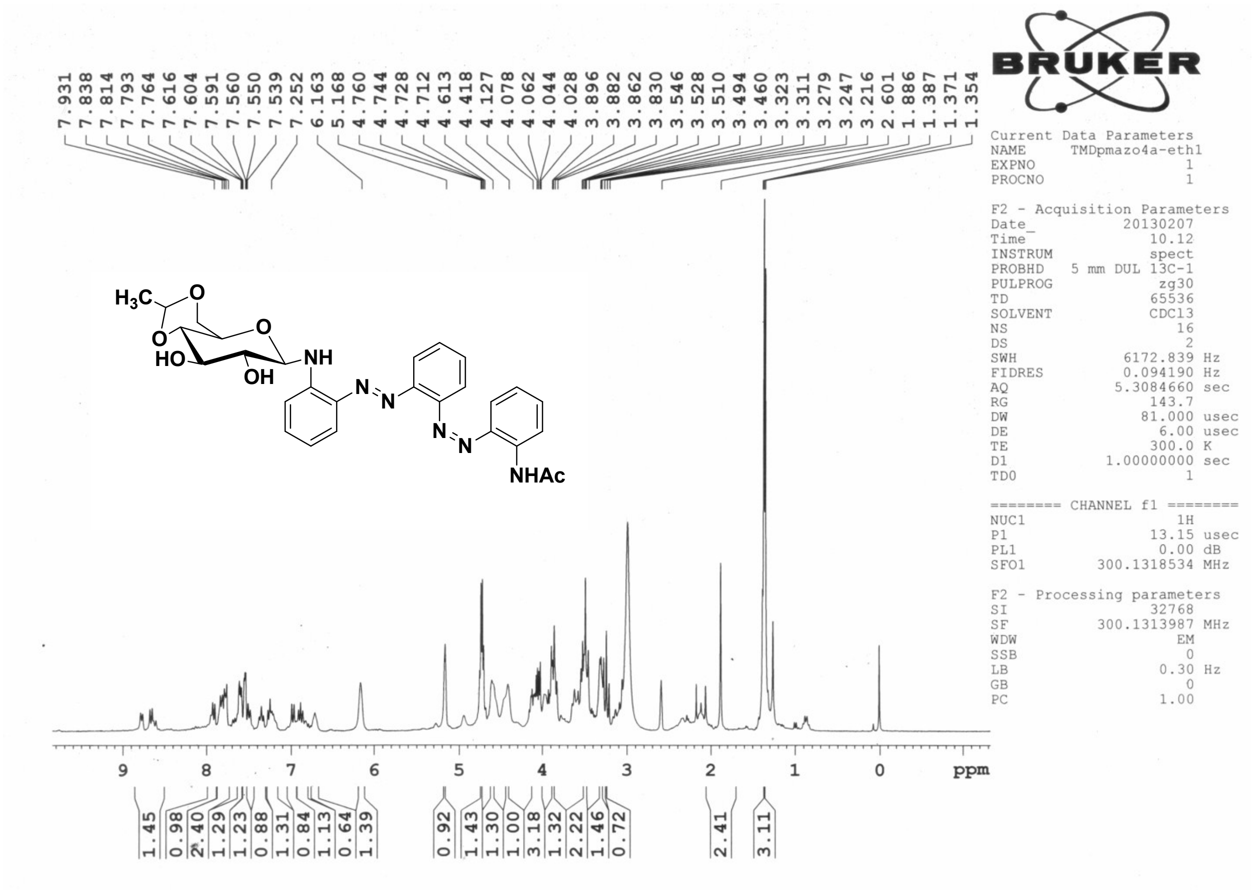

Figure S23. ^{13}C NMR Spectrum of compound 14 ($CDCl_3$(0.6mL) +DMSO- d_6 (0.1mL), 75MHz)

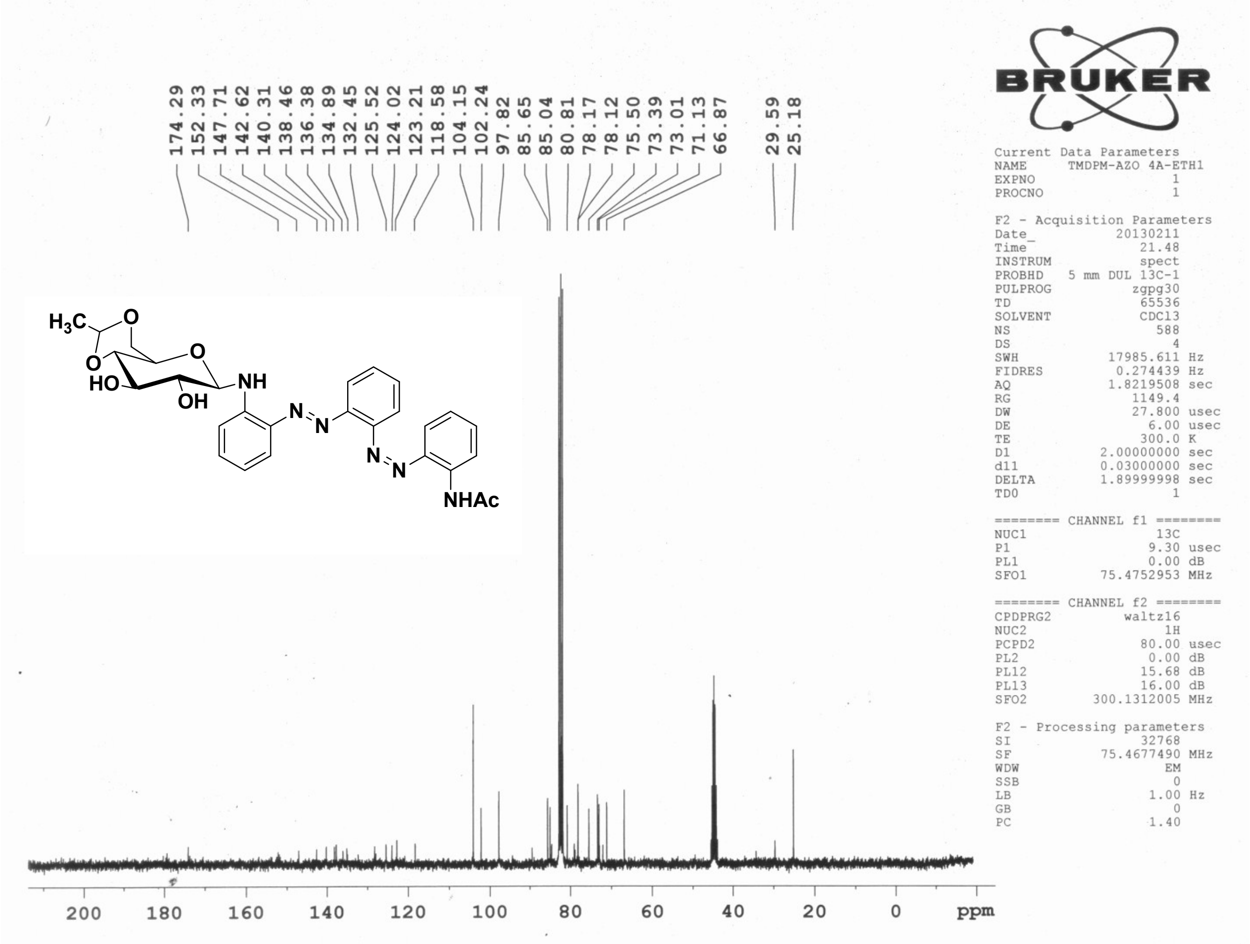

Figure S24. ^{1}H NMR Spectrum of compound 15($CDCl_3$(0.6mL) +DMSO- d_6 (0.1mL), 75MHz)

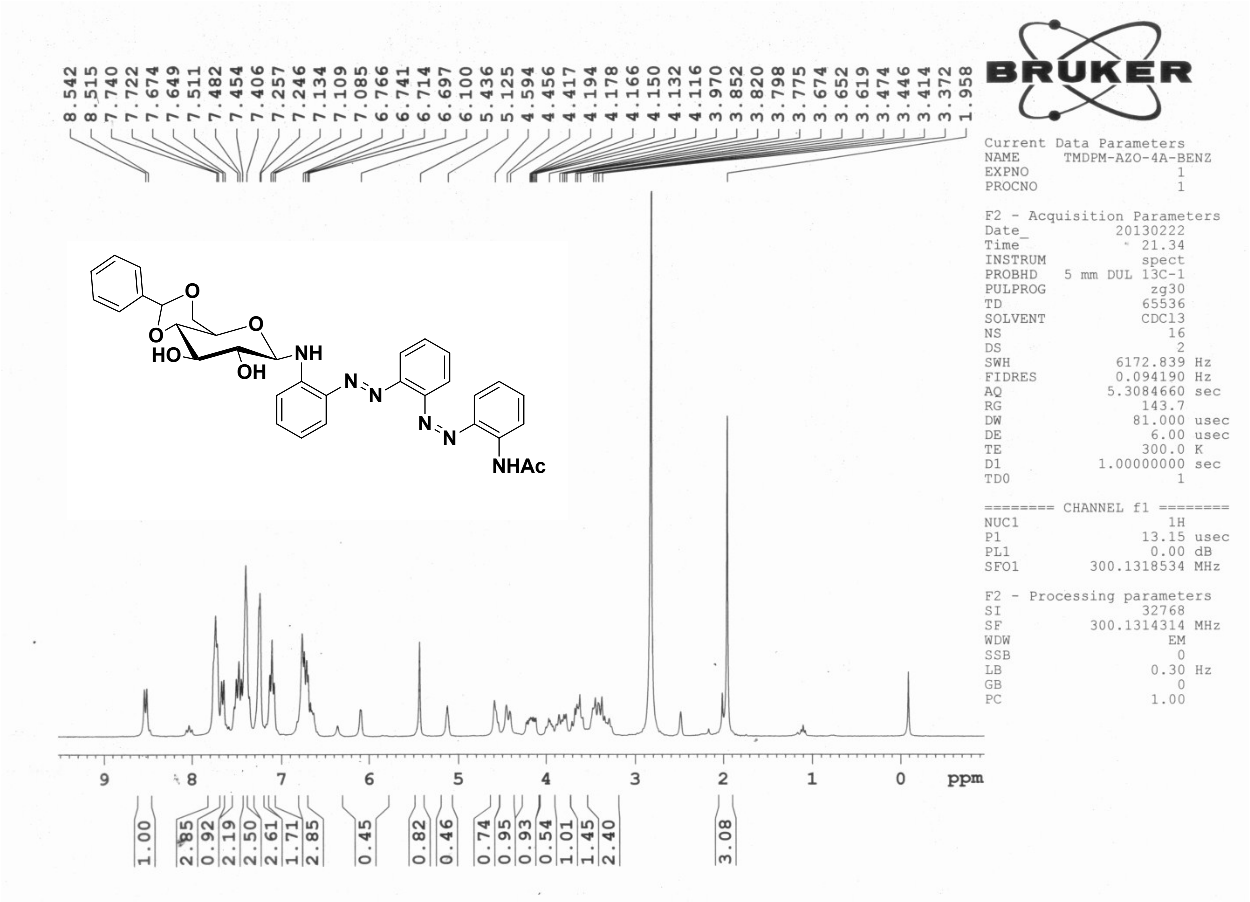

Figure S25. ^{13}C NMR Spectrum of compound 15 ($CDCl_3$(0.6mL) DMSO- d_6 (0.1mL), 75MHz)

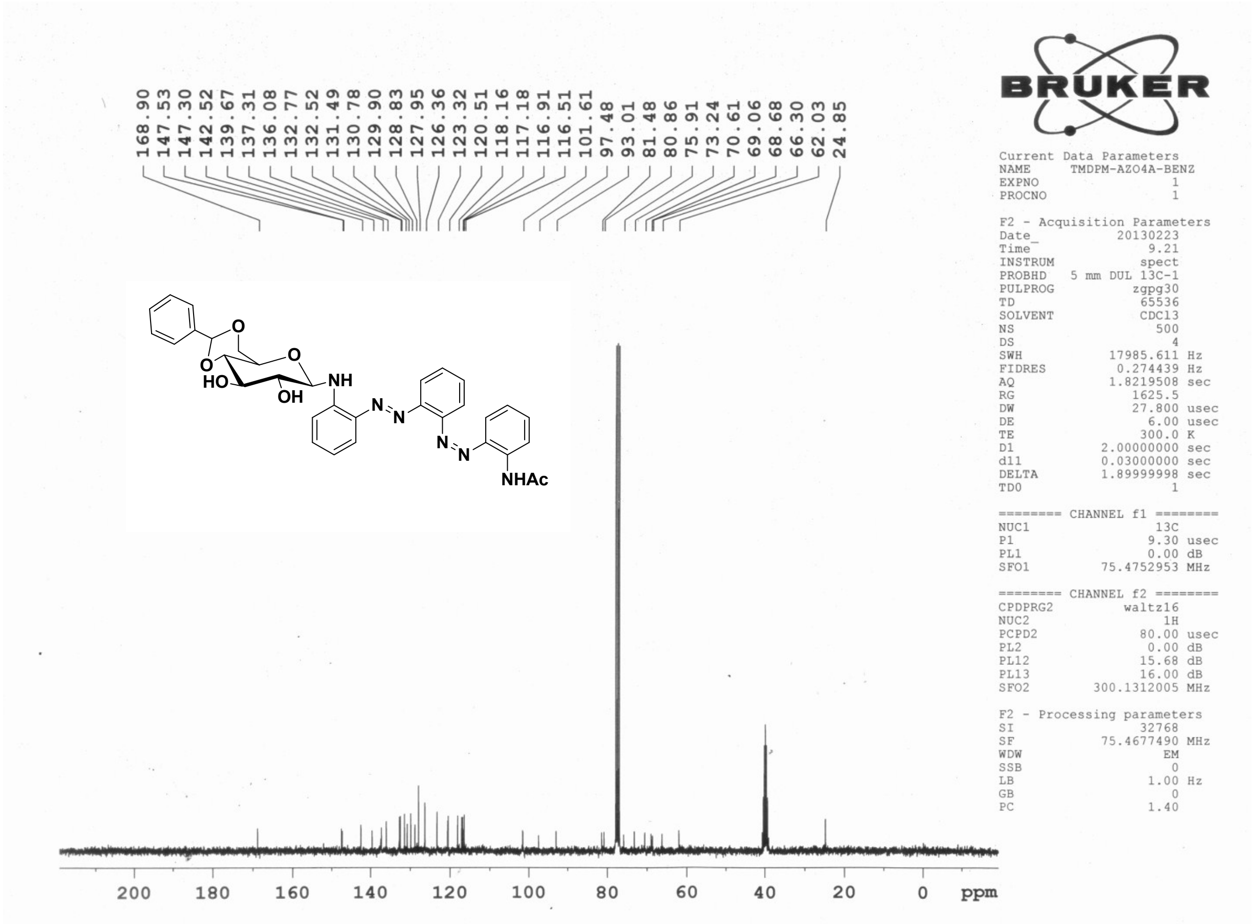

Supplement: RA-009-C9RA08033C-s001 [file RA-009-C9RA08033C-s001.pdf]
